# Chemical Constituents of *Oxytropis ochrocephala*

**DOI:** 10.3390/molecules30122489

**Published:** 2025-06-06

**Authors:** Guoli Li, Zhengyu Liu, Jiacheng Xie, Yunao Jin, Lei Wang, Hongying Yang, Yilin He, Tong Shen

**Affiliations:** Research Institute, School of Chemistry and Chemical Engineering, Lanzhou Jiaotong University, Lanzhou 730070, China; ligl@lzjtu.edu.cn (G.L.); liuzhengyu0011@163.com (Z.L.); xiejiacheng200011@163.com (J.X.); m13283861882@163.com (Y.J.); 11200606@stu.lzjtu.edu.cn (L.W.); yanghy@lzjtu.edu.cn (H.Y.)

**Keywords:** *Oxytropis ochrocephala*, phytochemistry, insecticidal activity

## Abstract

A new lignan (**1**) and a new phenolic glycoside (**2**), together with eighteen known compounds (**3**–**20**), were isolated from *Oxytropis ochrocephala*. Their structures were unambiguously elucidated by spectroscopic techniques (UV, IR, 1D and 2D NMR), and HR-ESI-MS analysis, as well as by comparison with the literature. The insecticidal activity of these compounds was evaluated against *Tetranychus urticae* Koch, and the results showed that compounds **3**, **9**, **15**, and **16** had a weak inhibitory effect at a concentration of 1 mg/mL after treatment for 24 h.

## 1. Introduction

**Figure 1:**
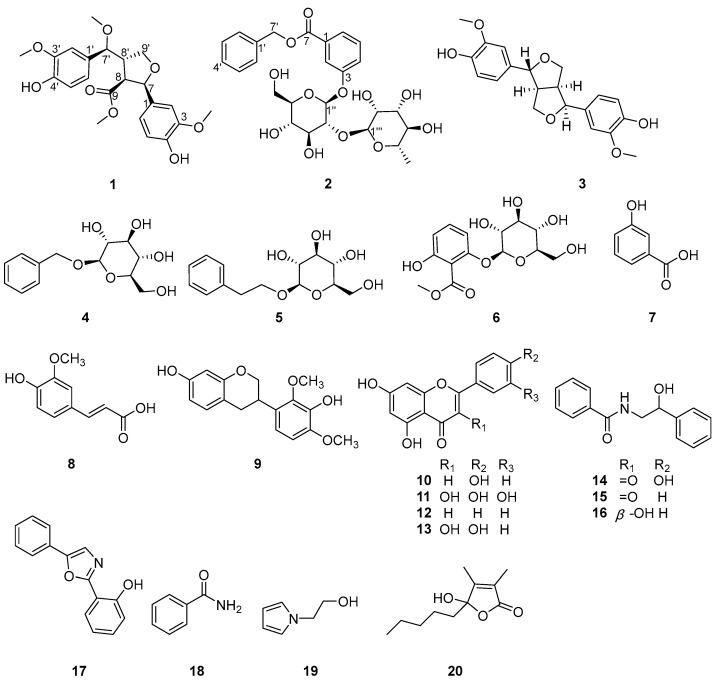
Structures of compounds **1**–**20**.

*Oxytropis ochrocephala* Bunge is a perennial herb belonging to the genus *Oxytropis* and the Fabaceae family. This plant primarily grows in the pastoral regions of Qinghai, Xizang, Gansu, and Sichuan provinces in China [1]. *O. ochrocephala* is a common poisonous plant and can cause chronic accumulation poisoning after being consumed by livestock, and thus is commonly known as “locoweed”. Moreover, the degradation of other grassland plants caused by salinization and rodents has facilitated the proliferation of *O. ochrocephala*. As a result, this plant has emerged as a dominant species, significantly disrupting the ecological balance. Interestingly, *O. ochrocephala* is also commonly found in arid steppes and desert areas and simultaneously serves as a vital agent in sand stabilization and desertification control, embodying a dual-natured ecological role [2]. *O. ochrocephala* is commonly employed to reduce heat by promoting detumescence, bolstering physical strength, and improving immune function [3,4]. Over the past forty years, nearly 300 compounds have been identified in *O. ochrocephala*, including alkaloids, flavonoids, triterpenoids, and saponins. These compounds demonstrate significant biological activities such as anti-tumor, anti-hepatitis B virus, insecticidal, antibacterial, and anti-hypoxia effects [5]. In our ongoing research to discover bioactive compounds from *O. ochrocephala*, we isolated and identified two new compounds (**1**, **2**) alongside eighteen known compounds (**3**–**20**) (Figure 1). The insecticidal properties of these compounds were evaluated.

## 2. Results

### 2.1. Structural Elucidation

Compound **1** was acquired as a white solid powder. Its molecular formula was determined to be C_22_H_26_O_8_ via HR-ESI-MS (*m*/*z* 441.1522 [M + Na]⁺, calcd for C_22_H_26_O_8_Na, 441.1520) and ^13^C NMR (Table 1). The IR spectrum of **1** exhibited characteristic absorption bands for the aromatic ring (1517 cm^−1^), carbonyl (1728 cm^−1^), and hydroxy (3419 cm^−1^). The ^1^H NMR spectrum displayed signals for two methines [δ_H_ 3.12 (1H, dd, *J* = 9.5, 8.4 Hz, H-8) and δ_H_ 3.01 (1H, dddd, *J* = 10.5, 9.5, 7.7, 7.5 Hz, H-8′)], two oxymethine protons [δ_H_ 5.09 (1H, d, *J* = 8.4 Hz, H-7) and δ_H_ 4.19 (1H, d, *J* = 10.5 Hz, H-7′)], one oxymethylene group [δ_H_ 3.54 (1H, dd, *J* = 9.2, 7.7 Hz, H-9a) and δ_H_ 3.71 (1H, dd, *J* = 9.2, 7.5 Hz, H-9b)], four methoxyl groups [δ_H_ 3.88 (3-OCH_3_), 3.90 (3′-OCH_3_), 3.05 (7′-OCH_3_), 3.74 (9-OCH_3_)], and six aromatic protons [δ_H_ 6.85 (1H, d, *J* = 1.9 Hz, H-2), 6.89 (1H, d, *J* = 8.0 Hz, H-5), 6.82 (1H, dd, *J* = 8.0, 1.9 Hz, H-6); 6.84 (1H, d, *J* = 1.9 Hz, H-2′), 6.87 (1H, d, *J* = 8.0 Hz, H-5′); 6.81 (1H, dd, *J* = 8.0, 1.9 Hz, H-6′)] (Table 1). The ^13^C NMR spectrum combined with the HSQC spectrum of **1** exhibited 22 carbon resonances corresponding to two 1,3,4-trisubstituted benzene rings (δ_C_ 132.4, 108.4, 146.7, 145.9, 114.4, 119.1; 131.5, 109.1, 147.1, 145.5, 114.4, 121.1), four methoxy groups (δ_C_ 56.1 × 3. 51.9), an ester carbonyl carbon (δ_C_ 173.1), an oxymethylene carbon (δ_C_ 70.5), two oxymethine carbons (δ_C_ 82.2, 84.0), and two methine carbons (δ_C_ 50.3, 54.4). These NMR data suggested that compound **1** is typical of a tetrahydrofuranoid-type lignan [6].

**Figure 2:**
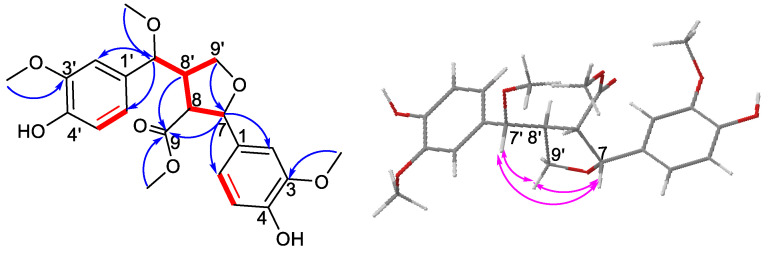
^1^H–^1^H COSY (red bold), HMBC (blue arrows), and NOESY (pink double arrows) correlations of compound **1**.

The planar structure of compound **1** was deduced from ^1^H–^1^H COSY cross-peaks and HMBC correlations. The cross-peaks of H-7/H-8/H-8′/H-7′(H-9′) in the ^1^H-^1^H COSY spectrum, combined with the HMBC correlations from H-9′ to C-7; H-7, H-8, H-8′ to C-9; H-7 to C-2/C-6; and H-7′ to C-2′/C-6′, confirmed a tetrahydrofuran ring and its substitutions as shown in Figure 2. The locations of the four methoxy groups at C-3′, C-3, C-7′, and C-9 were determined by the HMBC correlations from 3′-OCH_3_- to C-3′, 3-OCH_3_- to C-3, 7′-OCH_3_- to C-7′, H-7 to 7′-OCH_3_, and 9-OCH_3_- to C-9.

The relative configuration of compound **1** was determined by analysis of the coupling constants and NOESY spectrum (Figure 2). The NOESY correlations of H-7/H-9′α, H-7′/H-9′α suggested that these protons were co-facial and were randomly assigned as α-oriented. The large coupling constant of ^3^J_7′,8′_ = 10.5, ^3^J_8,8′_ = 9.4 Hz indicated a threo configuration. Consequently, H-8′ was β-oriented and H-8 was α-oriented. Thus, compound **1** was fully characterized as 7R*-methoxy-tanegool-9-methyl ester.

Compound **2** was obtained as a yellow oil. The HR-ESI-MS spectrum exhibited an ion peak at *m*/*z* 559.1785 [M + Na]^+^ (calcd for C_26_H_32_O_12_Na, 559.1786), from which the molecular formula of **2** was deduced to be C_26_H_32_O_12_, corresponding to 11 degrees of unsaturation. The IR spectrum indicated the presence of hydroxyl (3363 cm^−1^), ester carbonyl (1716 cm^−1^), aryl (1507 cm^−1^), and ether bond (1071 cm^−1^) groups. The ^1^H NMR (Table 1) showed signals for nine aromatic hydrogens (δ_H_ 7.74–7.30), two oxygenated methylenes (δ_H_ 5.36, 2H, d, *J* = 2.5 Hz, H-7′; 3.72, 3.86, m, H-6″), and two anomeric hydrogens (δ_H_ 5.10, 1H, d, *J* = 7.5 Hz, H-1″; δ_H_ 5.27, 1H, d, *J* = 1.5 Hz, H-1‴). The ^13^C NMR spectrum displayed twelve aromatic carbon signals (three quaternary and nine methine), one ester carbonyl carbon (δ_C_ 167.4, C-7), two oxygenated methylene carbons (δ_C_ 67.9, C-7′; 62.3, C-6″), and two anomeric carbons (δ_C_ 100.2, C-1″; 102.7, C-1‴).

**Figure 3:**
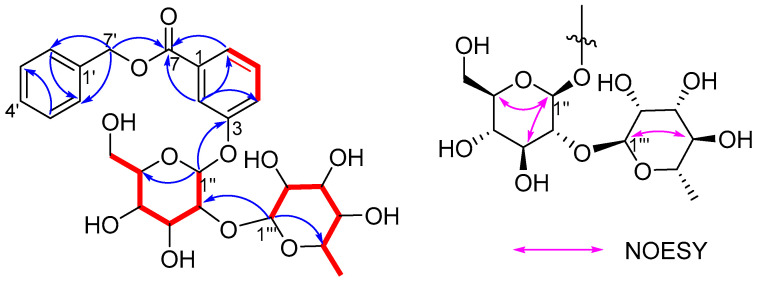
^1^H–^1^H COSY (red bold), HMBC (blue arrows), and NOESY correlations of compound **2**.

The ^1^H-^1^H COSY correlations of H-4/H-5/H-6, combined with the HMBC correlations from H-2 (*δ*_H_ 7.73) to C-4 (*δ*_C_ 122.0), C-6 (*δ*_C_ 124.3), indicated that there was a 1,3-disubstituted benzene ring. Although the proton signals of the other benzene ring overlap, it can be inferred to be a monosubstituted benzene based on the remaining five aromatic protons. The HMBC correlations from H-2, H-6 to C-7 and H-7′ to C-3, C-6, C-7 confirmed a benzyl 3-hydroxy-benzoate structure as shown in Figure 3 [7]. Furthermore, the ^1^H-^1^H COSY cross-peaks of H-1″/H-2″/H-3″/H-4″/H-5″/H-6″ and H-1‴/H-2‴/H-3‴/H-4‴/H-5‴/H-6‴ and the HMBC correlations from H-4 to C-2, C-6; H-5 to C-1, C-3; H-6 to C-2, C-4, C-7; H-2′ to C-4′, C-7′; H-3′ to C-1′, C-4′; H-4′ to C-2′, C-7′; H-7′ to C-7, C-1′, C-4′; H-1″ to C-3, C-5″; H-2″ to C-1″, C-3″, C-1‴; H-4″ to C-5″; H-1‴ to C-2″, C-2‴, C-5‴; H-4‴ to C-3‴, C-5‴, C-6‴; and H-6‴ to C-4‴, C-5‴, as well as comparison of their spectroscopic data [Table S1] with those reported in the literature, suggested that compound **2** has the same sugar chain of *α*-l-rhamnopyranosyl-(1 → 2)-*β*-d-glucopyranosyl as japonicanoside A [8], which is connected to C-3 in compound **2**. The relative configuration of the sugar chain was determined by the coupling constant (7.5 Hz) of H-1″/H-2″ and the characteristic signals of C-3‴ (*δ_C_* 72.2), C-5‴ (*δ_C_* 70.0), and C-6‴ (*δ_C_* 18.2) [9], which was confirmed by the NOESY correlations of H-3‴/H-5‴, H-1″/H-3″, and H-1″/H-5″. To further verify the absolute configuration of sugar, acid hydrolysis and derivatization were performed according to the methods described in the literature [10]. The LC analysis results [Figure S53] showed that the retention times of the glycosyl derivatives of compound **2** were consistent with those of the reference substances of D-glucose and L-rhamnose, respectively. Therefore, they were identified as *β*-D-glucose and *α*-L-rhamnose. Thus, the structure of **2** was assigned as benzyl 3-*O*-*α*-l-rhamnopyranosyl-(1 → 2)-*β*-d-glucopyranosyloxybenzoate.

In addition, the other known compounds were identified as pinoresinol (**3**) [11], benzyl *β*-d-glucopyranoside (**4**) [12], phenylethyl *β*-d-glucopyranoside (**5**) [13], methyl 2-*β*-d-glucopyranosyloxy-6-hydroxybenzoate (**6**) [14], 3-hydroxybenzoic acid (**7**) [15], ferulic acid (**8**) [16], 3′,7-dihydroxy-2′,4′-dimethoxyisoflavan (**9**) [17], apigenin (**10**) [18], quercetin (**11**) [18], chrysin (**12**) [19], kaempferol (**13**) [18], *N*-benzoyl-*β*-hydroxyphenylethylamine (**14**) [20], (*R*)-2-hydroxy-*N*-phenethyl-2-phenylacetamide (**15**) [21], *N*-benzoyl-phenylethylamine (**16**) [20], 2-(2′-hydroxyphenyl)-5-phenyloxazole (**17**) [13], benzamide (**18**) [22], 1-(2-hydroxyethyl)pyrrole (**19**) [23], and hydroxydihydrobovolide (**20**) [24], respectively, by comparing their spectroscopic data with those reported in the literature.

### 2.2. Biological Activity Analysis

*Tetranychus urticae* Koch belongs to the class Arachnida, family Tetranychidae, and genus *Tetranychus* [25]. This species is widely distributed globally and is characterized by its short generational cycle and high reproductive capacity. It inflicts damage on over 110 plant species across 32 families, making it one of the most detrimental mites in global agricultural and forestry production [26]. The prolonged use of chemical pesticides has led to increasingly prominent “3R” issues: residue, resistance, and resurgence. *T*. *urticae* has developed high resistance to more than 90 chemically active ingredients [27]. Therefore, the development of natural, highly effective, low-toxicity, and environmentally friendly bio-acaricides that are harmless to humans and livestock is of paramount importance. In light of this, we screened the contact toxicity activity of compounds **1**–**6** and **9**–**17** against *T*. *urticae*, with clofentezine used as a positive control. The results showed that compounds **3**, **9**, **15**, and **19** only had a weak inhibitory effect at a concentration of 1 mg/mL after treatment for 24 h (Table 2), while the other compounds did not exhibit any activity.

## 3. Materials and Methods

### 3.1. General Experimental Procedures

A polarimeter (Rudolph Research Analytical, Hackettstown, NJ, USA) was used to obtain optical rotation. The infrared (IR) data were recorded on a Bruker Tensor 27 spectrometer (Rudolph Research Analytical, USA). High-resolution electrospray ionization mass spectrometry (HR-ESI-MS) data were obtained using a Thermo Scientific LTQ-Orbitrap Elite ETD mass spectrometer (Thermo Fisher Scientific, Waltham, MA, USA). Nuclear magnetic resonance (^1^H, ^13^C, and 2D NMR) data were collected on a Bruker Avance NEO 500 MHz spectrometer (Bruker, Karlsruhe, Germany). Preparative HPLC separations were performed on a semi-preparative high-performance liquid chromatography Shimadzu LC-2030 (Shimadzu Corporation, Kyoto, Japan) equipped with a YMC-pack C18 (ODS) column (10 × 250 mm, 10 μm, Mitsubishi Chemical Corp., Tokyo, Japan). Macroporous resin, MCI resin (75–150 μm, Mitsubishi Chemical Corp., Tokyo, Japan), Sephadex LH-20 dextran gel (GE Healthcare Bio-Sciences, Uppsala, Sweden), reverse-phase silica gel PR-C18, and normal-phase silica gel (Qingdao Marine Chemical Co., Ltd., Qingdao, China) were used for column chromatography (CC). GF254 fluorescent silica gel (Qingdao Marine Chemical Co., Ltd.) was used for thin-layer chromatography (TLC).

### 3.2. Plant Materials

The whole plant of *Oxytropis ochrocephala* was collected in August 2020 in Xining City, Qinghai Province, China. Professor Que Sheng, School of Nationalities and Normal Education, Qinghai Normal University, taxonomically identified the plant from a sample of a whole flowering plant. The plant specimen was deposited at the Natural Medicine Development Research Institute, Lanzhou Jiaotong University, with accession number HHJD-20200930.

### 3.3. Extraction and Isolation

The whole plant material of *O. ochrocephala* (15.8 kg) underwent triple percolation with 95% ethanol (EtOH, 3 × 50 L). The obtained crude extract (1.25 kg) was dissolved in warm water (1 L) at 50 °C. The solution was acidified to a pH range of 1–3 by the addition of a 2% hydrochloric acid (HCl) solution and was subsequently extracted three times with dichloromethane (CH_2_Cl_2_, 3 × 1 L), yielding an acidic extract weighing 330.12 g. This acidic extract was fractionated on a macroporous resin column and eluted with a water/methanol (H_2_O/MeOH) gradient (100:0 to 0:100, *v*/*v*), resulting in the formation of five fractions: F1 (0% MeOH, 5.65 g); F2 (30% MeOH, 9.81 g); F3 (50% MeOH, 16.02 g); F4 (80% MeOH, 87.53 g); and F5 (100% MeOH, 71.7 g). An additional fraction, F6 (124.72 g), was obtained through elution with acetone. Fractions F3 and F4 were chosen for further separation owing to their high compound diversity, as revealed by preliminary analysis. Fraction F3 (16.02 g) was chromatographed on a silica gel column and eluted with a CH_2_Cl_2_/MeOH gradient (100:0 to 0:100, *v*/*v*) to obtain sub-fractions F3.1–F3.5. Sub-fraction F3.3 (2.06 g) was chromatographed on a silica gel column, eluted with a gradient CH_2_Cl_2_/MeOH (100:0 to 0:100, *v*/*v*), and produced sub-fractions F3.3.1–F3.3.8, which were further purified by silica gel (petroleum ether/ethyl acetate, 2:1, *v*/*v*) and then underwent HPLC (MeOH/H_2_O, 60:40, *v*/*v*, flow rate: 2 mL/min), yielding compounds **7** (t*_R_* = 49.02 min, 5.2 mg), **15** (t*_R_* = 9.19 min, 5.8 mg), and **18** (t*_R_* = 16.28 min, 4.4 mg). F3.5 (4.36 g) was subjected to chromatographic separation on a reversed-phase C18 (RP-18) column and eluted with a gradient of H_2_O/MeOH ranging from 100:0 to 0:100 (*v*/*v*). This process led to the generation of sub-fractions F3.5.1–F3.5.11. These fractions were purified using Sephadex LH-20 (MeOH/CH_2_Cl_2_, 1:1, *v*/*v*), respectively, resulting in the isolation of compound **19** (2.6 mg), compound **8** (56.2 mg), and compound **5** (25.1 mg). Fraction F4 (87.53 g) was processed under the same chromatographic conditions as fraction F3, yielding sub-fractions F4.1–F4.10. F4.4 (6.79 g) was chromatographed on a silica gel column with a CH_2_Cl_2_/MeOH gradient from 100:0 to 0:100 (*v*/*v*), providing sub-fractions F4.4.1–F4.4.5. Sub-fraction F4.4.3 (1.43 g) was separated on an RP-18 column using a H_2_O/MeOH gradient of 100:0 to 0:100 (*v*/*v*), yielding sub-fractions F4.4.3.1–F4.4.3.8. Sub-fraction F4.4.3.2 (0.25 g) was purified on silica gel with a petroleum ether/ethyl acetate (30:1, *v*/*v*) eluent, yielding compounds **6** (3.5 mg), **11** (3.6 mg), **12** (2.3 mg), and **17** (12.3 mg). Sub-fraction F4.4.4 (1.76 g) was chromatographed on silica gel with a petroleum ether/ethyl acetate (10:1, *v*/*v*) eluent and further purified by semi-preparation HPLC with a mobile phase of MeOH/H_2_O (60:40, *v*/*v*, flow rate: 2 mL/min), leading to the isolation of compounds **2** (*t*_R_ = 22.31 min, 20.2 mg), **4** (*t*_R_ = 31.73 min, 4.2 mg), **14** (*t*_R_ = 39.24 min, 2.6 mg), and **16** (*t*_R_ = 24.62 min, 9.3 mg). Sub-fraction F4.5 (9.92 g) was separated on a silica gel column with a CH_2_Cl_2_/MeOH gradient (100:0 to 0:100, *v*/*v*), resulting in the generation of sub-fractions F4.5.1–F4.5.8. F4.5.3 (1.24 g) was chromatographed on a reversed-phase C18 (RP-18) column with a H_2_O/MeOH (70:30, *v*/*v*) and further purified on a Sephadex LH-20 column using a CH_2_Cl_2_/MeOH (1:1, *v*/*v*), leading to the isolation of compound **9** (3.3 mg) and compound **13** (5.0 mg). F4.5.4 (0.46 g) was purified on silica gel with a petroleum ether/ethyl acetate (10:1, *v*/*v*), yielding compound **10** (3.7 mg) and compound **20** (6.2 mg). F4.5.6 (0.37 g) was purified by silica gel chromatography using petroleum ether/ethyl acetate (5:1, *v*/*v*), followed by semi-preparation high-performance liquid chromatography (HPLC) with MeOH/H_2_O (70:30, *v*/*v*, flow rate: 2 mL/min), yielding compound **1** (*t*_R_ = 26.39 min, 4.2 mg) and compound **3** (*t*_R_ = 19.62 min, 3.6 mg).

### 3.4. Biology Assay

The insecticidal activity against the two-spotted spider mite, *Tetranychus urticae* Koch, was assessed using the capillary micro-droplet technique [27]. The compounds isolated from *O. ochrocephala* were dissolved in dimethyl sulfoxide (DMSO) (10 mg/mL) and prepared in three concentration levels: 10 mg/mL, 2 mg/mL, and 1 mg/mL. Healthy, wingless individuals of equal size were chosen for the tests. The test solutions were applied to the dorsal thorax of the mites using a capillary tube. Each concentration was evaluated in triplicate with 20 mites treated in each replicate. Concurrently, a control group was also set up. The treated mites were placed in 12 cm diameter Petri dishes lined with moist filter paper. These dishes were sealed with plastic wrap, punctured for air circulation, labeled, and kept at room temperature for incubation. The survival of the mites was checked every 24 h, and the number of dead mites was recorded. During this observation period, distilled water was added as necessary to keep the filter paper moist. Mortality was determined by a complete lack of movement when the legs and antennae were brushed. The mortality rate (%) was calculated as (number of dead mites/total number of mites) × 100, while the corrected mortality rate (%) was derived from [(control group survival rate − treatment group survival rate)/control group survival rate] × 100.

### 3.5. Compound Characterization

Compound **1**: white amorphous solid; ${\left[\alpha \right]}_{D}^{20.7}$−25.0 (*c* 0.01, MeOH); IR (KBr) *ν*_max_ 3419, 2928, 1728, 1517, 1274 cm^−1^; ^1^H (CDCl_3_, 500 MHz) and ^13^C NMR (CDCl_3_, 125 MHz), see Table 1; HR-ESI-MS *m*/*z* 441.1522 [M + Na]^+^ (calcd for C_22_H_26_O_8_Na, 441.1520).

Compound **2**: yellow oil; ${\left[\alpha \right]}_{D}^{20.7}$+32.0 (*c* 0.01, MeOH); IR (KBr) *ν*_max_ 3363, 1716, 1507, 1071 cm^−1^; ^1^H (CDCl_3_, 500 MHz) and ^13^C NMR (CDCl_3_, 125 MHz), see Table 1; HR-ESI-MS *m/z* 559.1785 [M + Na]^+^ (calcd for C_26_H_32_O_12_Na, 559.1786).

## 4. Conclusions

In summary, our intensive phytochemical investigation of the plant *O. ochrocephala* identified twenty compounds, including a novel lignan (**1**), a phenolic glycoside (**2**), and eighteen known compounds: a lignan (**3**), five phenols (**4**–**8**), five flavonoids (**9**–**13**), six alkaloids (**14**–**19**), and another type compound (**20**). Their structures were unambiguously elucidated by spectroscopic techniques and HR-ESI-MS analysis, as well as by comparison with the literature. Insecticidal activity against *Tetranychus urticae* indicated that these compounds showed a weak inhibitory effect.

## Figures and Tables

**Table 1:** ^1^H NMR (500 MHz) and ^13^C NMR (126 MHz) data of compounds **1** and **2** (*δ* in ppm, *J* in Hz).

Title 1	1 ^a^			2 ^b^	
No.	*δ*_H_	*δ*_C_	No.	*δ*_H_	*δ*_C_
1		132.4	1		132.8
2	6.85, d (1.9)	108.4	2	7.73, t (1.5)	118.4
3		146.7	3		158.7
4		145.9	4	7.32, m	122.0
5	6.89, d (8.0)	114.4	5	7.43, m	130.8
6	6.82, dd (8.0, 1.9)	119.1	6	7.71, dt (1.5, 7.5)	124.3
7	5.09, d (8.4)	84.0	7		167.4
8	3.12, dd (9.5, 8.4)	54.4	1′		137.6
9		173.1	2′, 6′	2H, 7.48, m	129.2
			3′, 5′	2H, 7.41, m	129.7
OMe-3	3.88, s	56.1	4′	7.36, m	129.3
OMe-9	3.74, s	51.9	7′	2H, 5.36, d (2.5)	67.9
1′		131.5	1″	5.10, d (7.5)	100.2
2′	6.84, d (1.9)	109.1	2″	3.68, m	79.5
3′		147.1	3″	3.61, m	79.1
4′		145.5	4″	3.45, m	71.3
5′	6.87, d (8.0)	114.4	5″	3.45, m	78.0
6′	6.81, dd (8.0, 1.9)	121.1	6″	2H, 3.72, 3.86, m	62.3
7′	4.19, d (10.5)	82.2	1‴	5.27, d (1.5)	102.7
8′	3.01, dddd (10.5, 9.5, 7.7, 7.5)	50.3	2‴	3.95, m	72.2
9′	3.54, dd (9.2, 7.7)	70.5	3‴	3.60, m	72.2
	3.71, dd (9.2, 7.5)		4‴	3.40, m	74.0
OMe-3′	3.90, s	56.1	5‴	3.97, m	70.0
OMe-7′	3.05, s	56.1	6‴	3H, 1.28, d (6.5)	18.2

^a^ Tested in CDCl_3_; ^b^ tested in CD_3_OD.

**Table 2:** The contact toxicity activity of the active compounds **3**, **9**, **15**, and **19** against *T. urticae*.

Com.	Mortality Rate/%	Corrected Mortality Rate/%
**3**	26.6	26.6
**9**	33.3	33.3
**15**	40.0	40.0
**19**	33.3	33.3
Clofentezine	90.0	90.0

## Data Availability

The original contributions presented in this study are included in the article/Supplementary Material. Further inquiries can be directed to the corresponding author(s).

## Supplementary Materials

The following supporting information can be downloaded at: https://www.mdpi.com/article/10.3390/molecules30122489/s1, The 1D and 2D NMR, IR spectra, and HR-ESI-MS data for **1** and **2**, and the 1D NMR of **3**–**20** (PDF).
