# Supplementary material for: Chemical Constituents of Oxytropis ochrocephala
Source: Molecules. 2025 Jun 6;30(12):2489. doi: 10.3390/molecules30122489 (PMC12196023; doi:10.3390/molecules30122489)
Supplement: Supplementary file 1 [file molecules-30-02489-s001.zip › molecules-3659368-supplementary.pdf]

SUPPLEMENTARY MATERIAL

Chemical constituents of *Oxytropis ochrocephala*

Guo-Li Li [1], Zheng-Yu Liu [1], Jia-Cheng Xie [1] , Yun-Ao Jin [1] , Lei Wang [1] , Hong-Ying yang, Yi-Lin He [1*] and Tong Shen [1,*]

[1] *Research Institute; School of Chemistry and Chemical Engineering, Lanzhou Jiaotong University, Lanzhou 730070, China;*

*Corresponding author: S_tong28@163.com; heyl@lzjtu.edu.cn

Figure S1 ^{1}H NMR spectrum of **1** in $CDCl_3$ (500 MHz)....4
Figure S2 ^{13}C NMR spectrum of **1** in $CDCl_3$ (126 MHz)....4
Figure S3 HSQC spectrum of **1** in $CDCl_3$ (126 MHz)....5
Figure S4 HMBC spectrum of **1** in $CDCl_3$ (126 MHz)....5
Figure S6 NOESY spectrum of **1** in (500 MHz)....6
Figure S7 HR-ESIMS spectrum of **1**....7
Figure S8 IR spectrum of **1**....7
Figure S9 ^{1}H NMR spectrum of **2** in CD_3OD (500 MHz)....8
Figure S10 ^{13}C NMR spectrum of **2** in CD_3OD (126 MHz)....8
Figure S11 HSQC spectrum of **2** in CD_3OD (126 MHz)....9
Figure S12 HMBC spectrum of **2** in CD_3OD (126 MHz)....9
Figure S13 ^{1}H-^{1}H COSY spectrum of **2** in CD_3OD (500 MHz)....10
Figure S14 NOESY spectrum of **2** in CD_3OD (500 MHz)....10
Figure S15 HR-ESIMS spectrum of **2**....11
Figure S16 IR spectrum of **2**....11
Figure S17 ^{1}H NMR spectrum of **3** in $CDCl_3$ (500 MHz)....12
Figure S18 ^{13}C NMR spectrum of **3** in $CDCl_3$ (126 MHz)....12
Figure S19 ^{1}H NMR spectrum of **4** in CD_3OD (500 MHz)....13
Figure S20 ^{13}C NMR spectrum of **4** in CD_3OD (126 MHz)....13
Figure S21 ^{1}H NMR spectrum of **5** in CD_3OD (500 MHz)....14
Figure S22 ^{13}C NMR spectrum of **5** in CD_3OD (126 MHz)....14
Figure S23 ^{1}H NMR spectrum of **6** in CD_3OD (500 MHz)....15
Figure S24 ^{13}C NMR spectrum of **6** in CD_3OD (126 MHz)....15
Figure S25 ^{1}H NMR spectrum of **7** in CD_3OD (500 MHz)....16
Figure S26 ^{13}C NMR spectrum of **7** in CD_3OD (126 MHz)....16
Figure S27 ^{1}H NMR spectrum of **8** in CD_3OD (500 MHz)....17
Figure S28 ^{13}C NMR spectrum of **8** in CD_3OD (126 MHz)....17
Figure S29 ^{1}H NMR spectrum of **9** in $CDCl_3$ (500 MHz)....18
Figure S30 ^{13}C NMR spectrum of **9** in $CDCl_3$ (126 MHz)....18
Figure S31 ^{1}H NMR spectrum of **10** in DMSO-*d6* (500 MHz)....19
Figure S32 ^{13}C NMR spectrum of **10** in DMSO-*d6* (126 MHz)....19
Figure S33 ^{1}H NMR spectrum of **11** in DMSO-*d6* (500 MHz)....20
Figure S34 ^{13}C NMR spectrum of **11** in DMSO-*d6* (126 MHz)....20
Figure S35 ^{1}H NMR spectrum of **12** in DMSO-*d6* (500 MHz)....21
Figure S36 ^{13}C NMR spectrum of **12** in DMSO-*d6* (126 MHz)....21
Figure S37 ^{1}H NMR spectrum of **13** in CD_3OD (500 MHz)....22
Figure S38 ^{13}C NMR spectrum of **13** in CD_3OD (126 MHz)....22
Figure S39 ^{1}H NMR spectrum of **14** in CD_3OD (500 MHz)....23
Figure S40 ^{13}C NMR spectrum of **14** in CD_3OD (126 MHz)....23
Figure S41 ^{1}H NMR spectrum of **15** in $CDCl_3$ (500 MHz)....24
Figure S42 ^{13}C NMR spectrum of **15** in $CDCl_3$ (126 MHz)....24
Figure S43 ^{1}H NMR spectrum of **16** in $CDCl_3$ (500 MHz)....25
Figure S44 ^{13}C NMR spectrum of **16** in $CDCl_3$ (126 MHz)....25

Figure S45 ^{1}H NMR spectrum of **17** in Acetone-*d6* (500 MHz) 26
Figure S46 ^{13}C NMR spectrum of **17** in Acetone-*d6* (126 MHz) 26
Figure S47 ^{1}H NMR spectrum of **18** in $CDCl_3$ (500 MHz) 27
Figure S48 ^{13}C NMR spectrum of **18** in $CDCl_3$ (126 MHz) 27
Figure S49 ^{1}H NMR spectrum of **19** in CD_3OD (500 MHz) 28
Figure S50 ^{13}C NMR spectrum of **19** in CD_3OD (126 MHz) 28
Figure S51 ^{1}H NMR spectrum of **20** in CD_3OD (500 MHz) 29
Figure S52^{13}C NMR spectrum of **20** in CD_3OD (126 MHz) 29
Table S1 ^{1}H NMR (500 MHz) and ^{13}C NMR (126 MHz) data of compounds **2** and japonicanoside A (δ in ppm, J in Hz). 30
Figure S53 HPLC analyses of the authentic samples (A) D-glucose, (B) L-rhamnose, and (C) the hydrolysates and derivatives of compound **2** 31
Figure S54 The process of Extraction and Separation 32

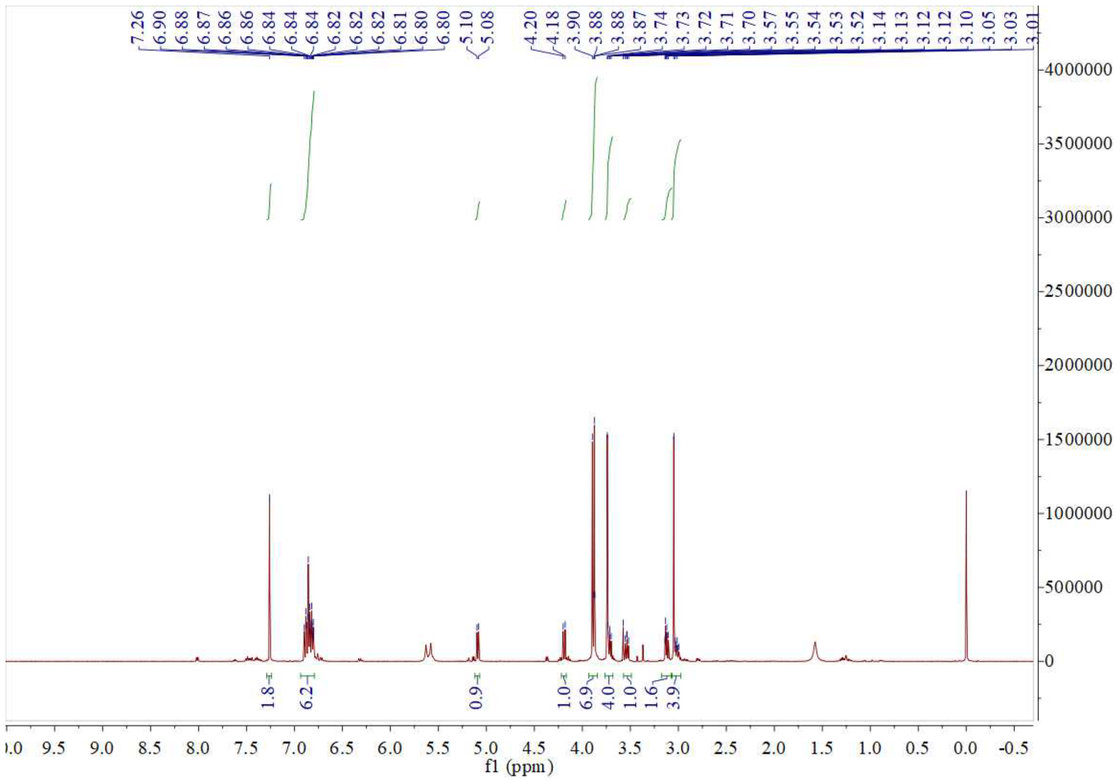

Figure S1 ^{1}H NMR spectrum of **1** in $CDCl_3$ (500 MHz)

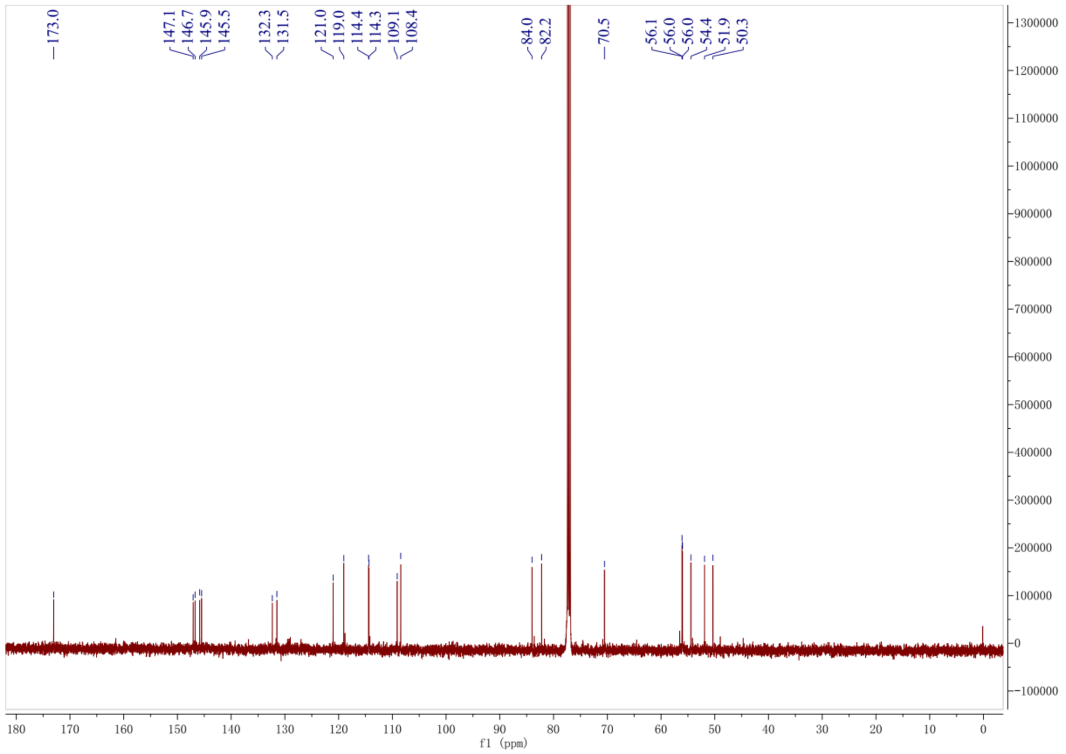

Figure S2 ^{13}C NMR spectrum of **1** in $CDCl_3$ (126 MHz)

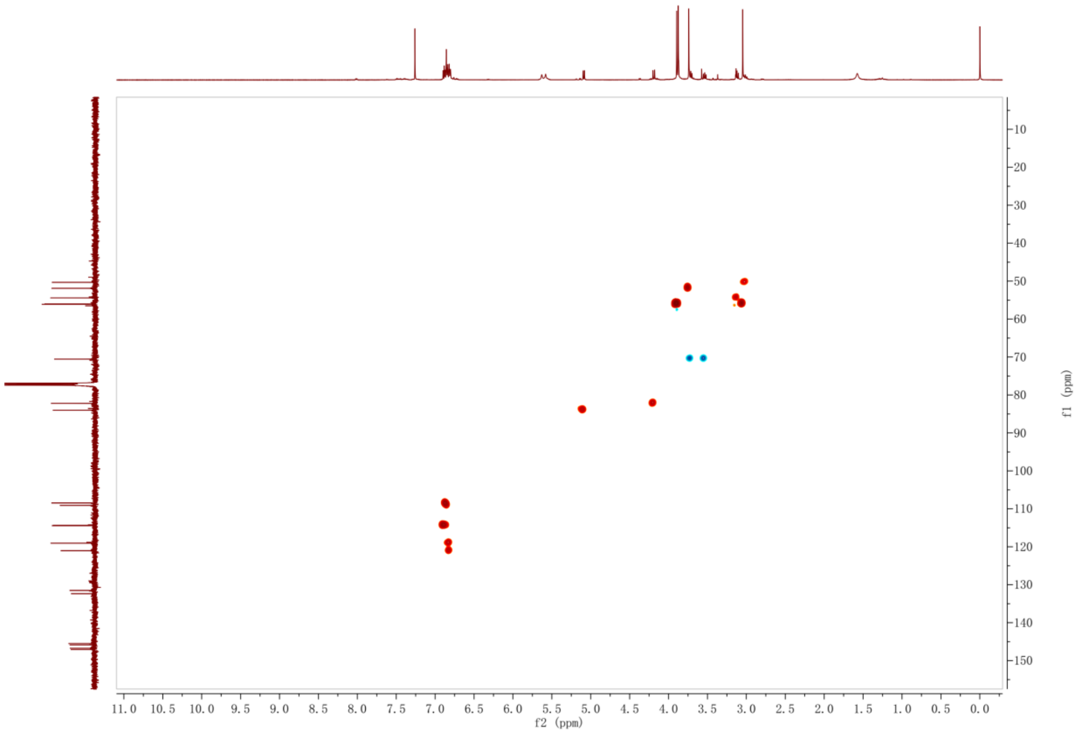

Figure S3 HSQC spectrum of **1** in $CDCl_3$ (126 MHz)

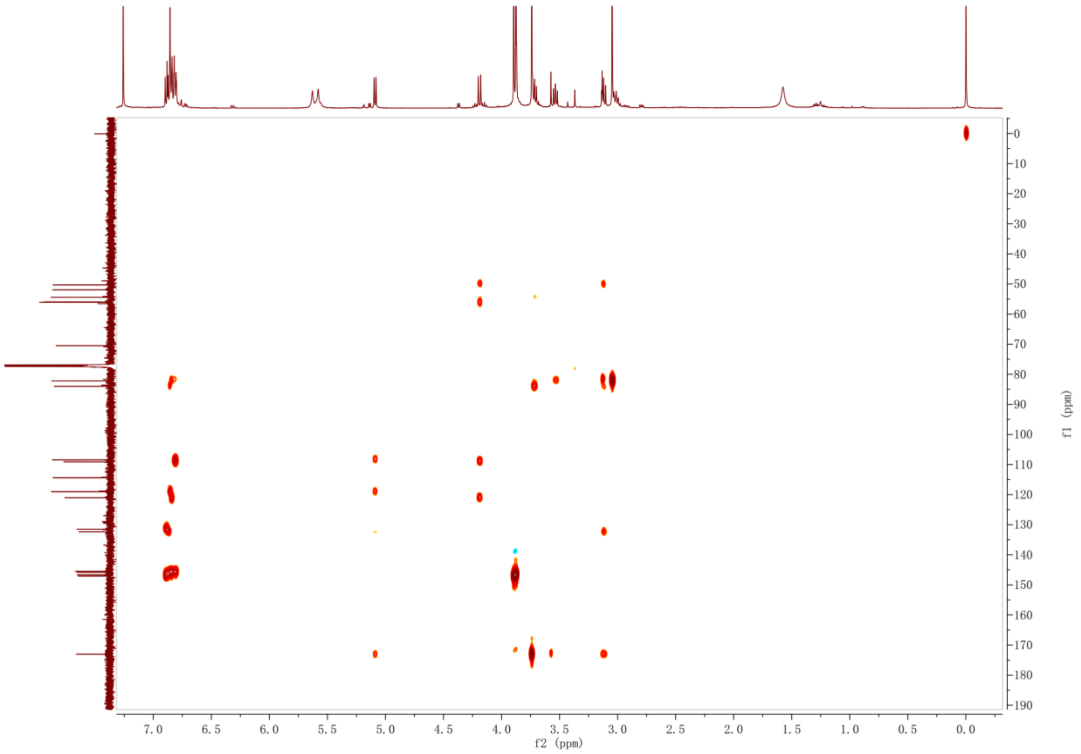

Figure S4 HMBC spectrum of **1** in $CDCl_3$ (126 MHz)

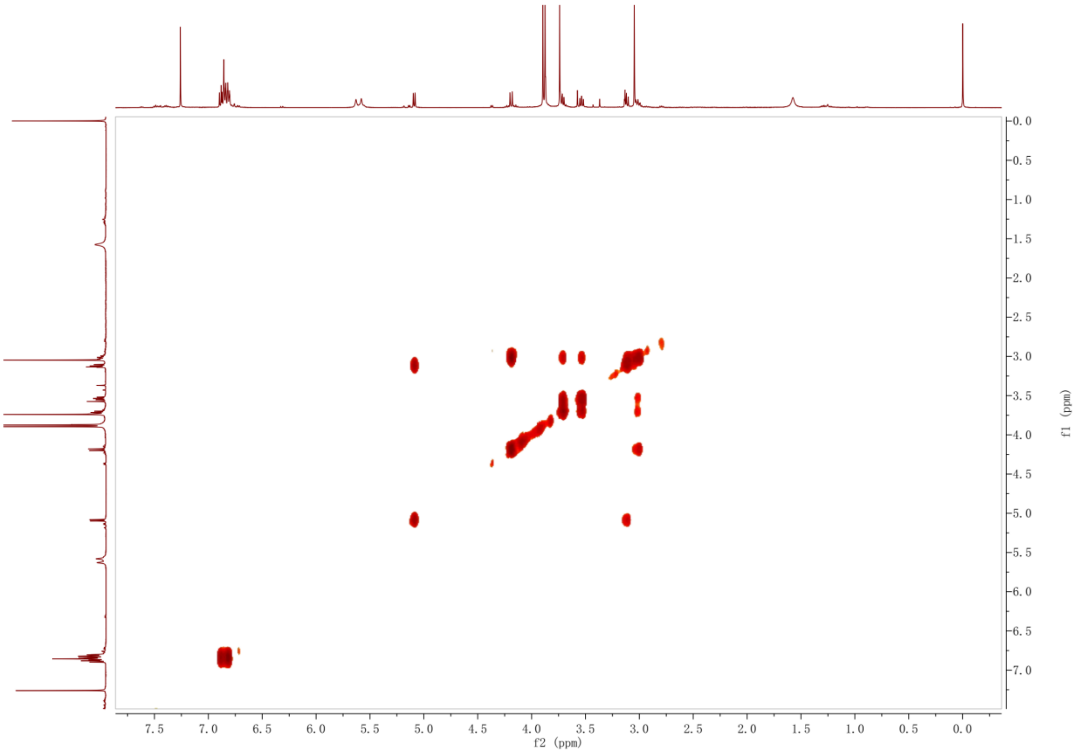

Figure S5 ^{1}H-^{1}H COSY spectrum of **1** in $CDCl_3$ (500 MHz)

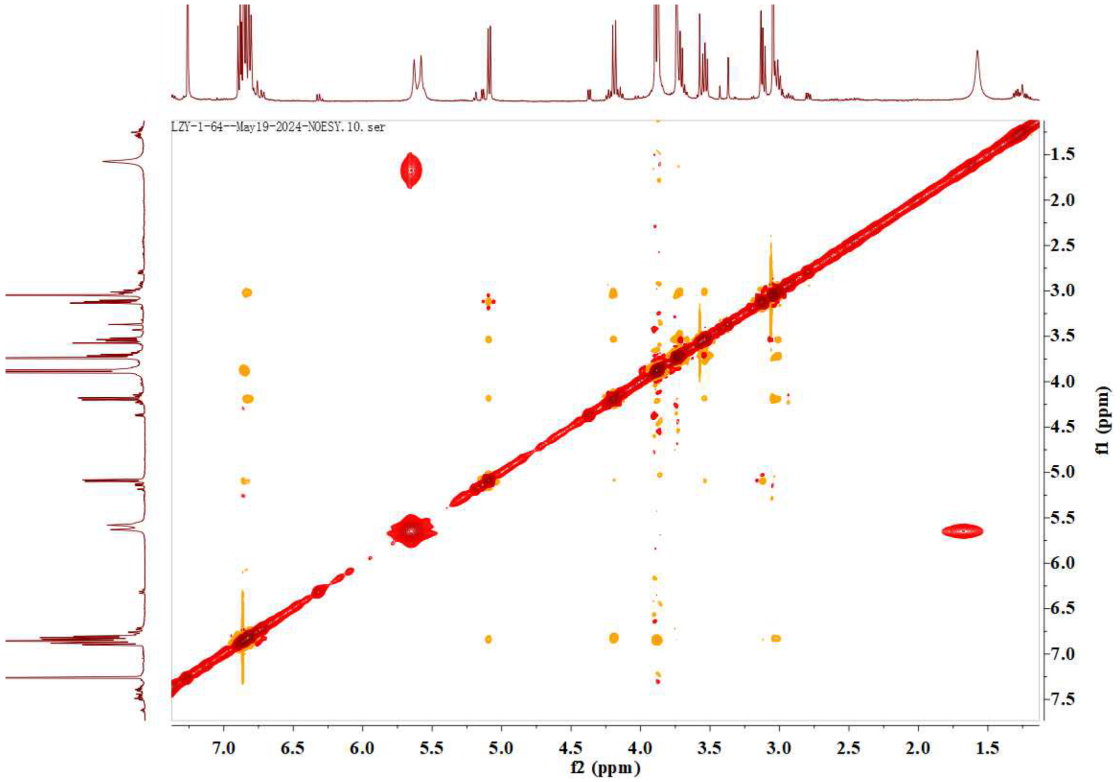

Figure S6 NOESY spectrum of **1** in (500 MHz).

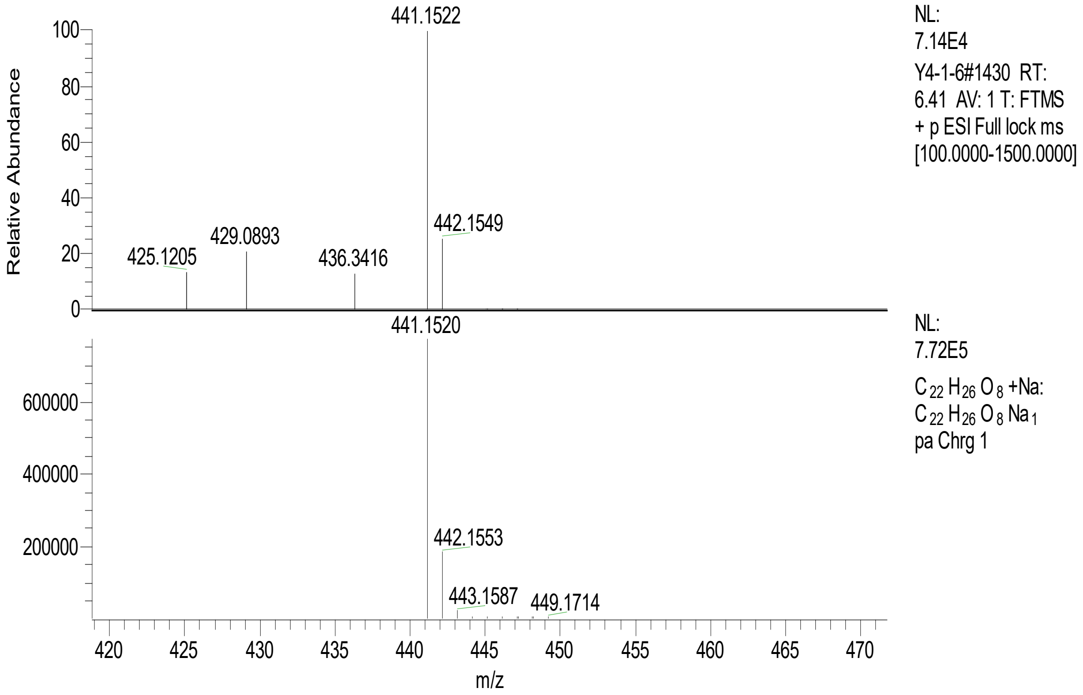

Figure S7 HR-ESIMS spectrum of **1**

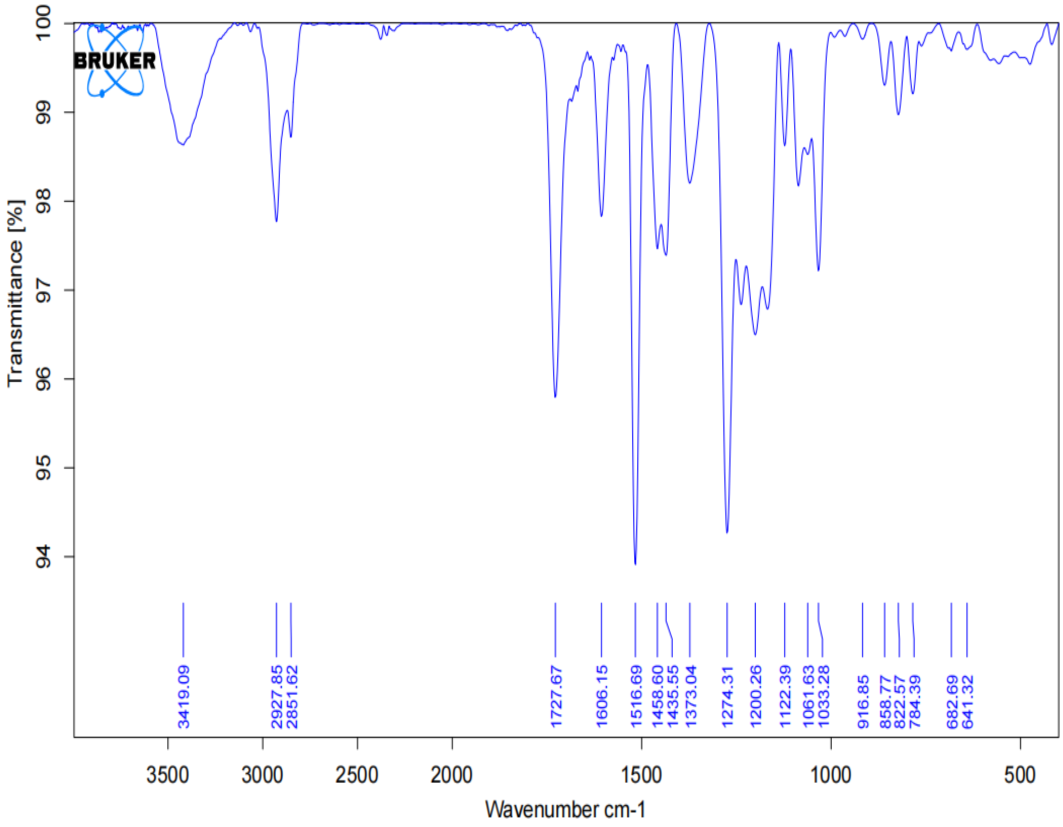

Figure S8 IR spectrum of **1**

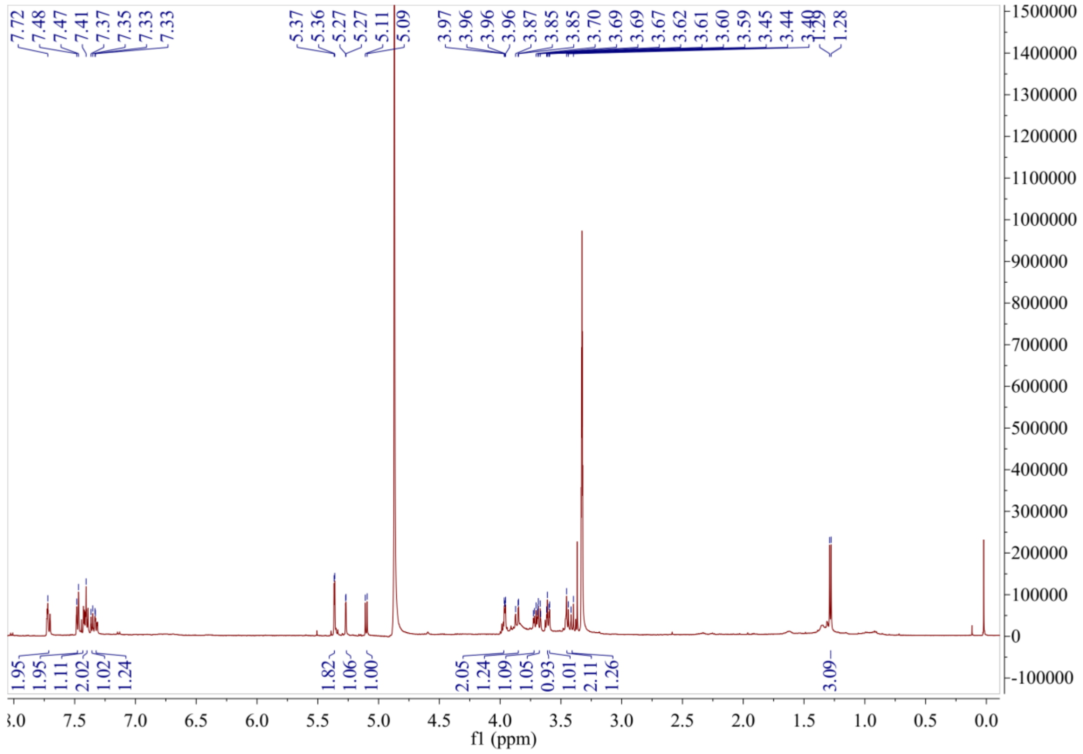

Figure S9 ^{1}H NMR spectrum of **2** in CD_3OD (500 MHz)

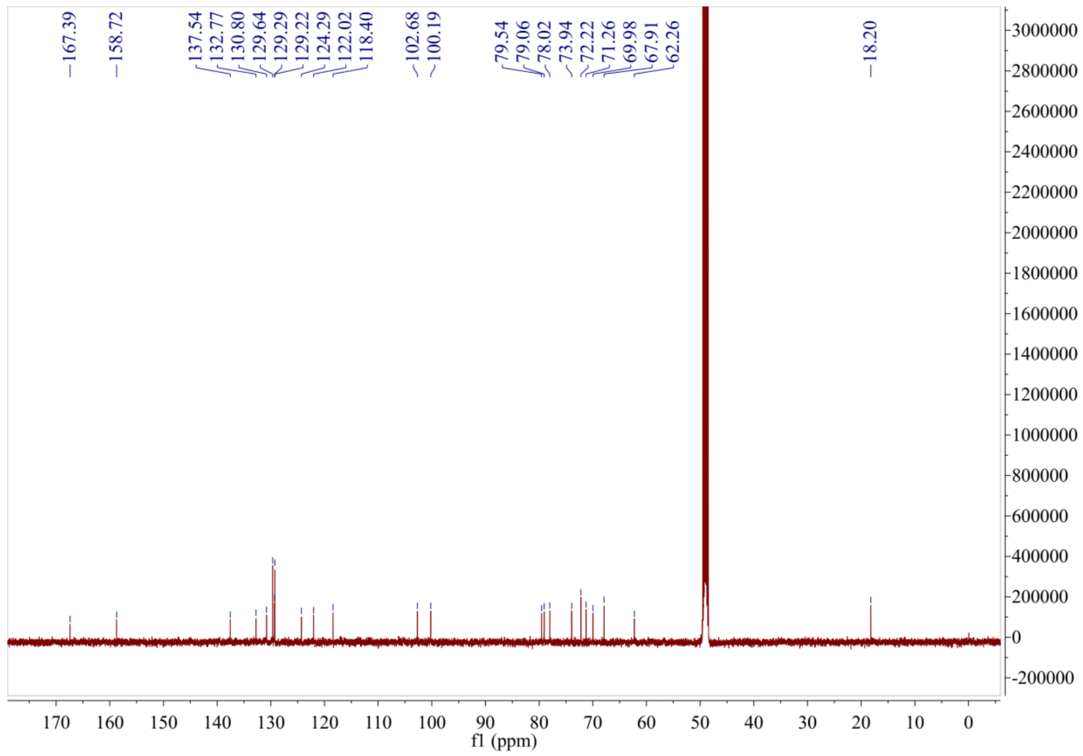

Figure S10 ^{13}C NMR spectrum of **2** in CD_3OD (126 MHz)

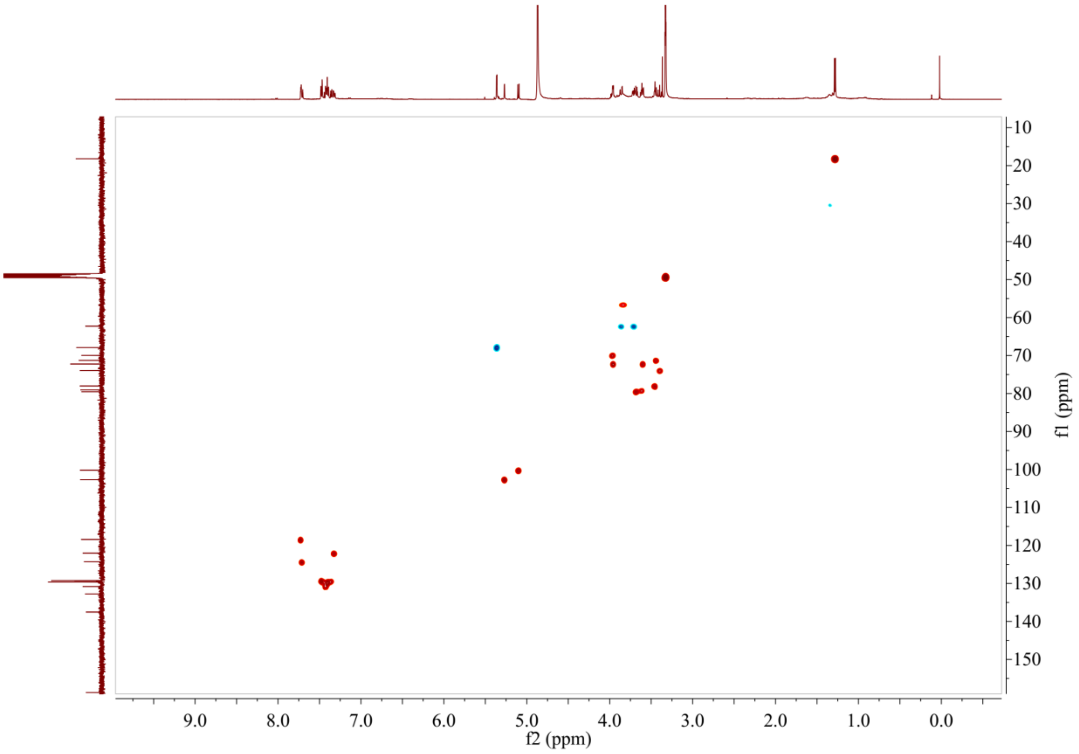

Figure S11 HSQC spectrum of **2** in CD_3OD (126 MHz)

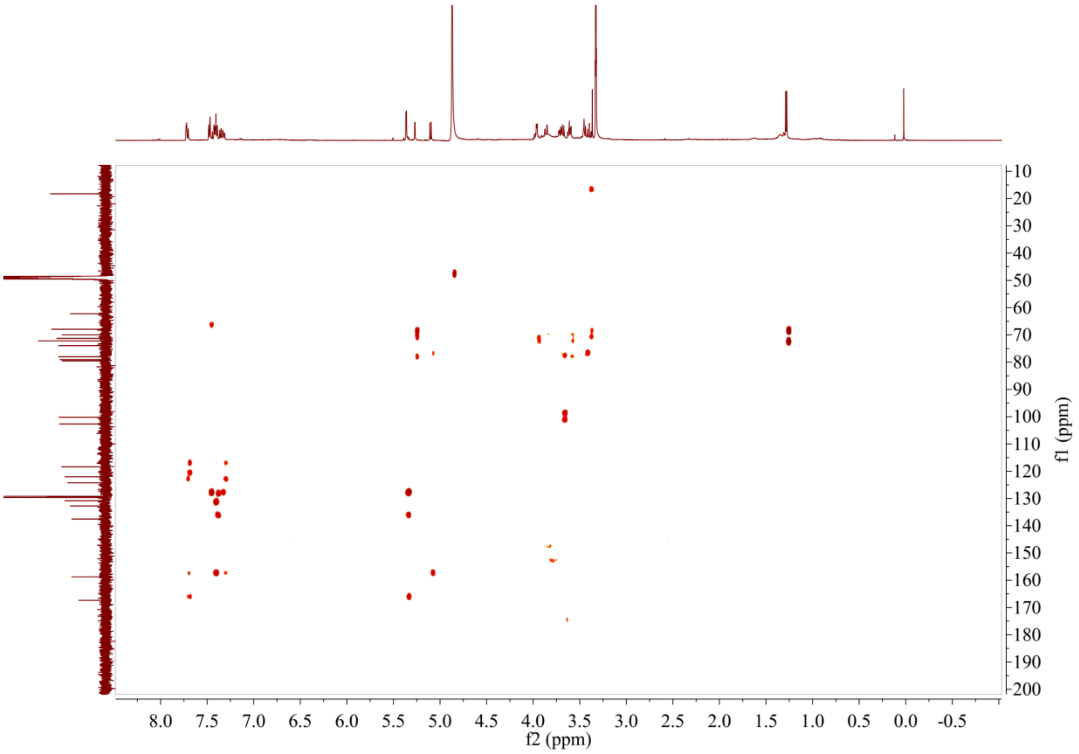

Figure S12 HMBC spectrum of **2** in CD_3OD (126 MHz)

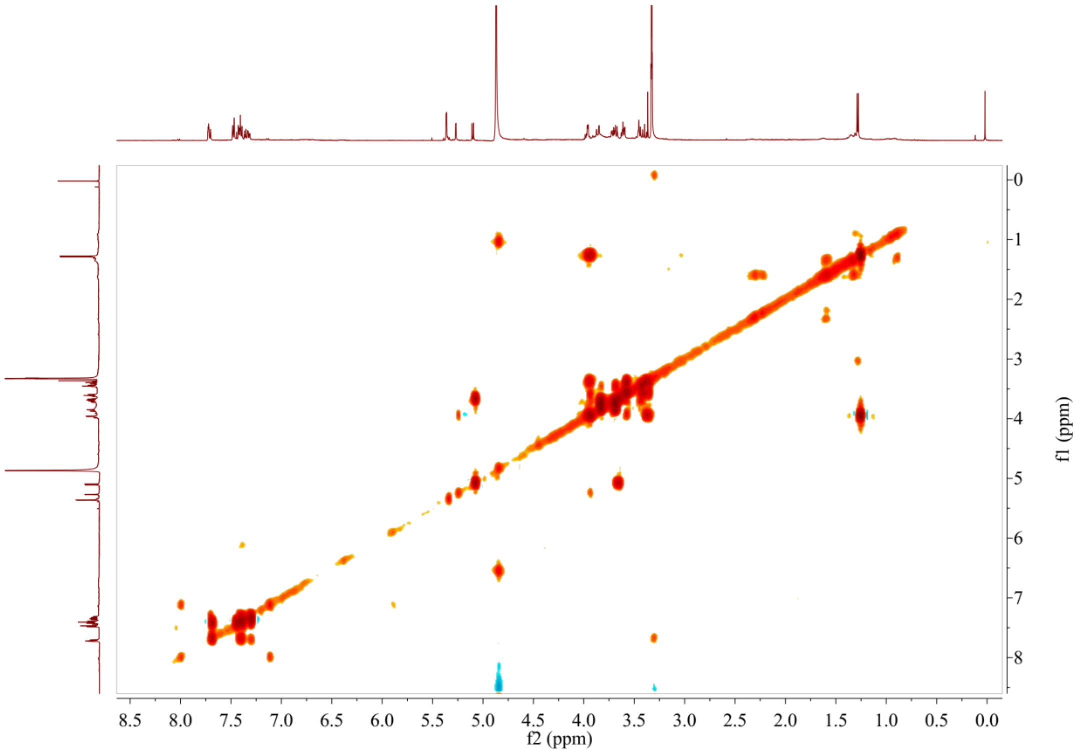

Figure S13 ^{1}H-^{1}H COSY spectrum of **2** in CD_3OD (500 MHz)

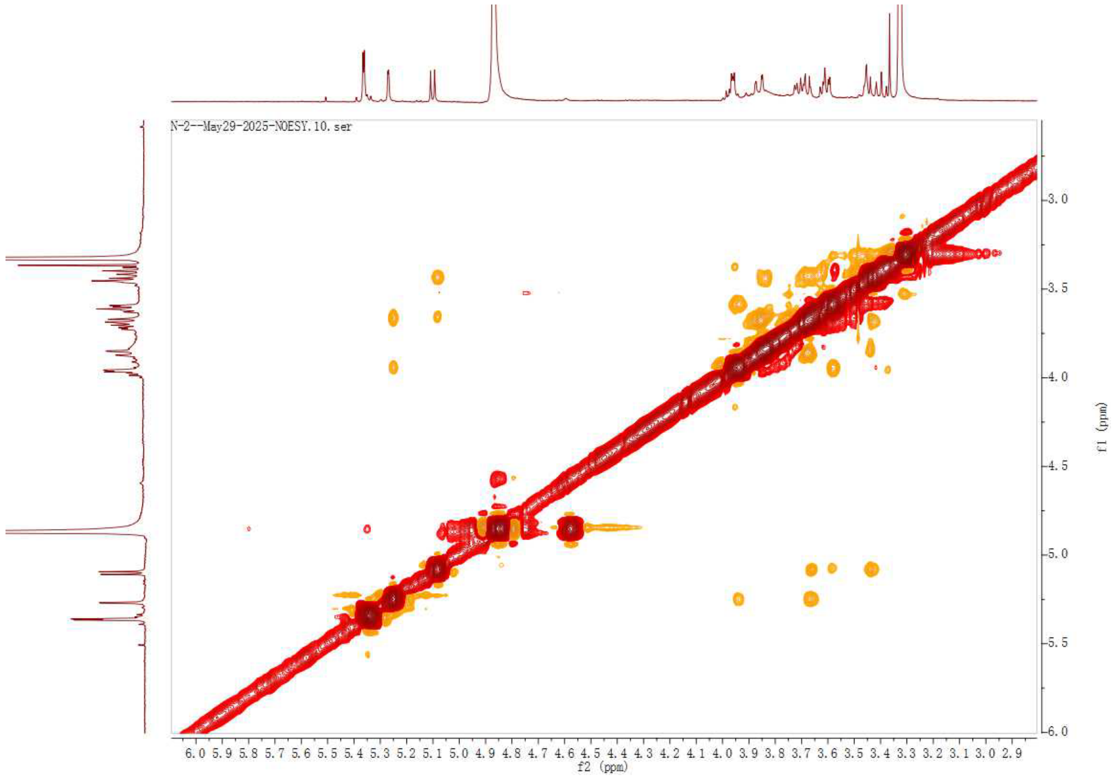

Figure S14 NOESY spectrum of **2** in CD_3OD (500 MHz)

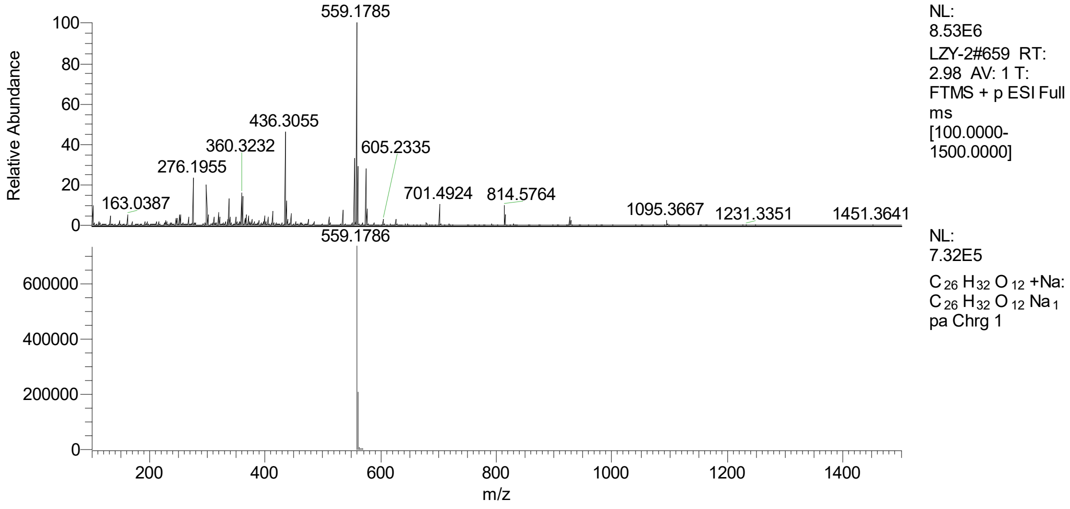

Figure S15 HR-ESIMS spectrum of **2**

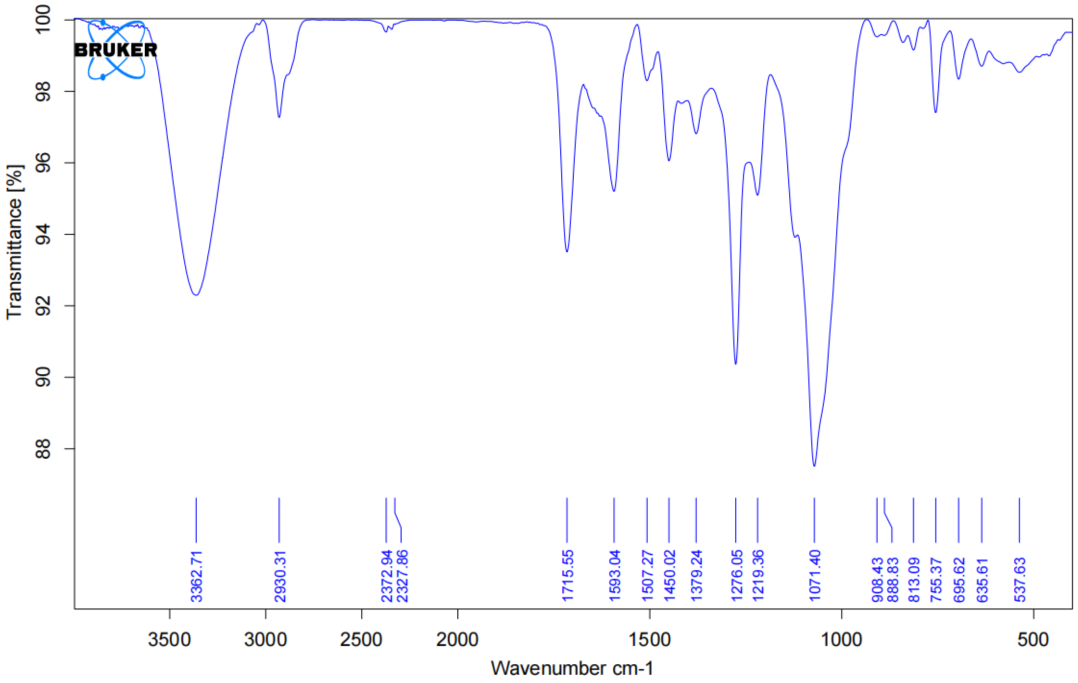

Figure S16 IR spectrum of **2**

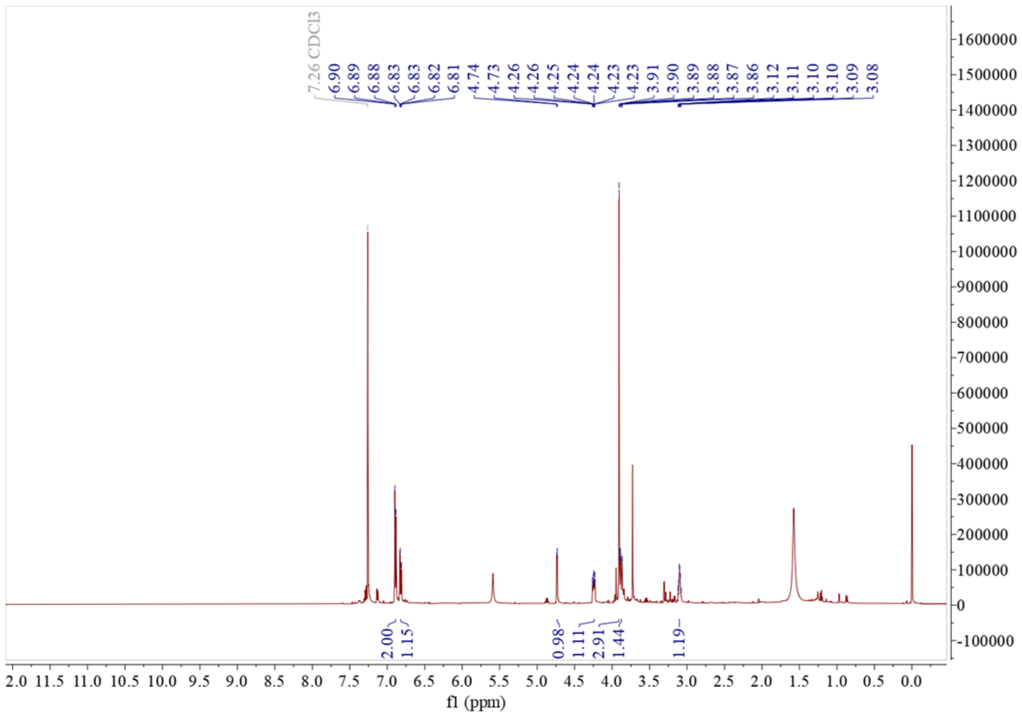

Figure S17 ^{1}H NMR spectrum of **3** in $CDCl_3$ (500 MHz)

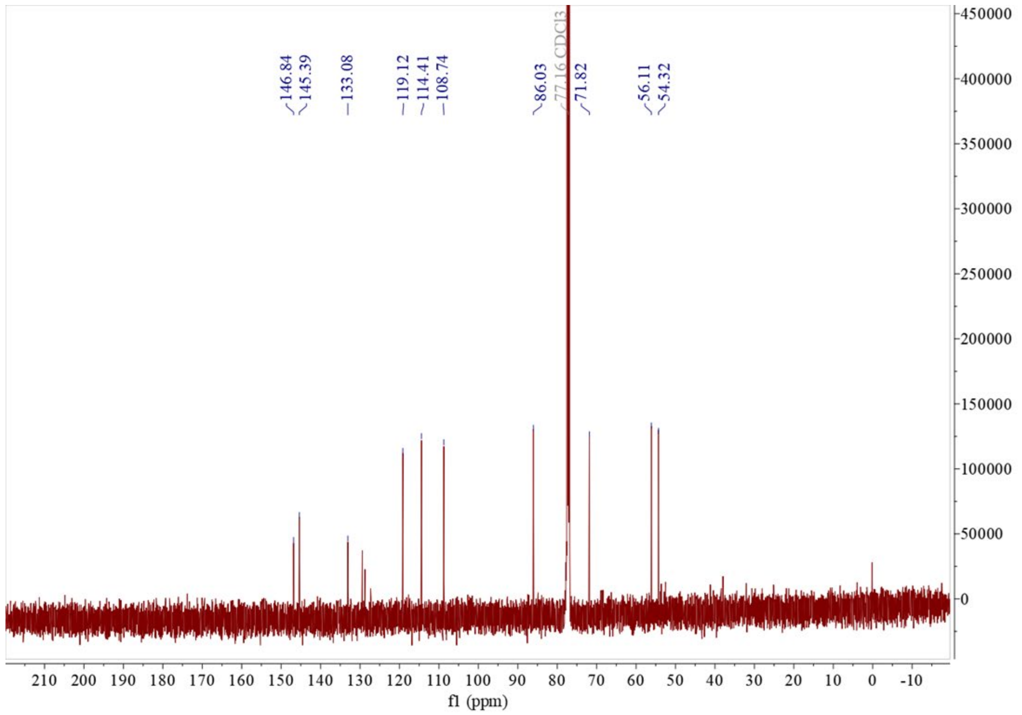

Figure S18 ^{13}C NMR spectrum of **3** in $CDCl_3$ (126 MHz)

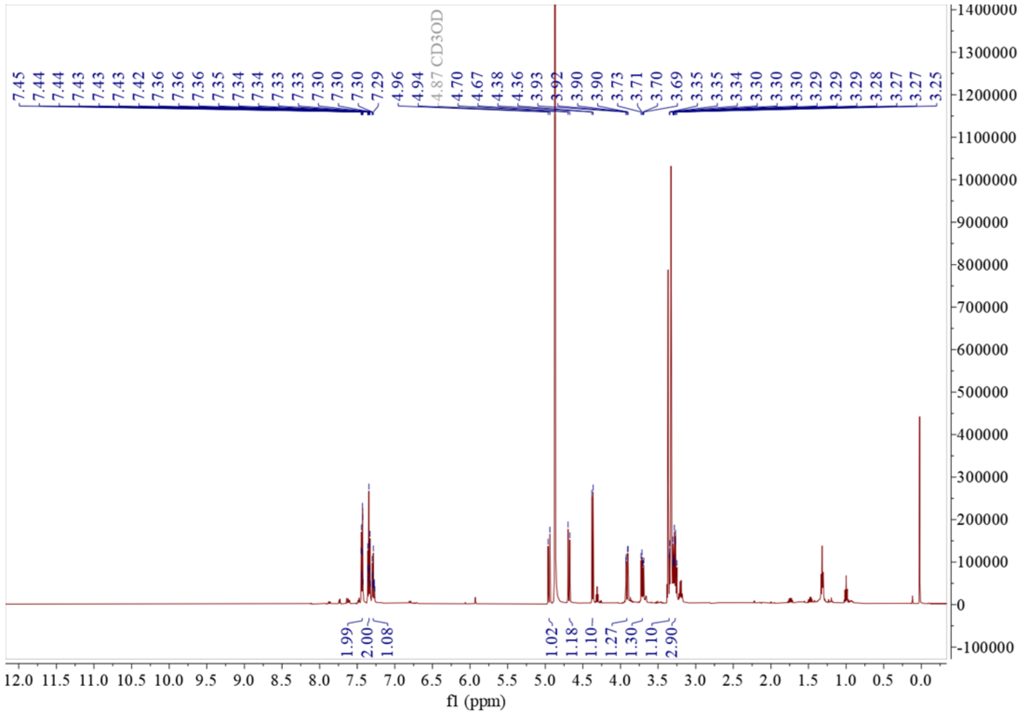

Figure S19 ^{1}H NMR spectrum of **4** in CD_3OD (500 MHz)

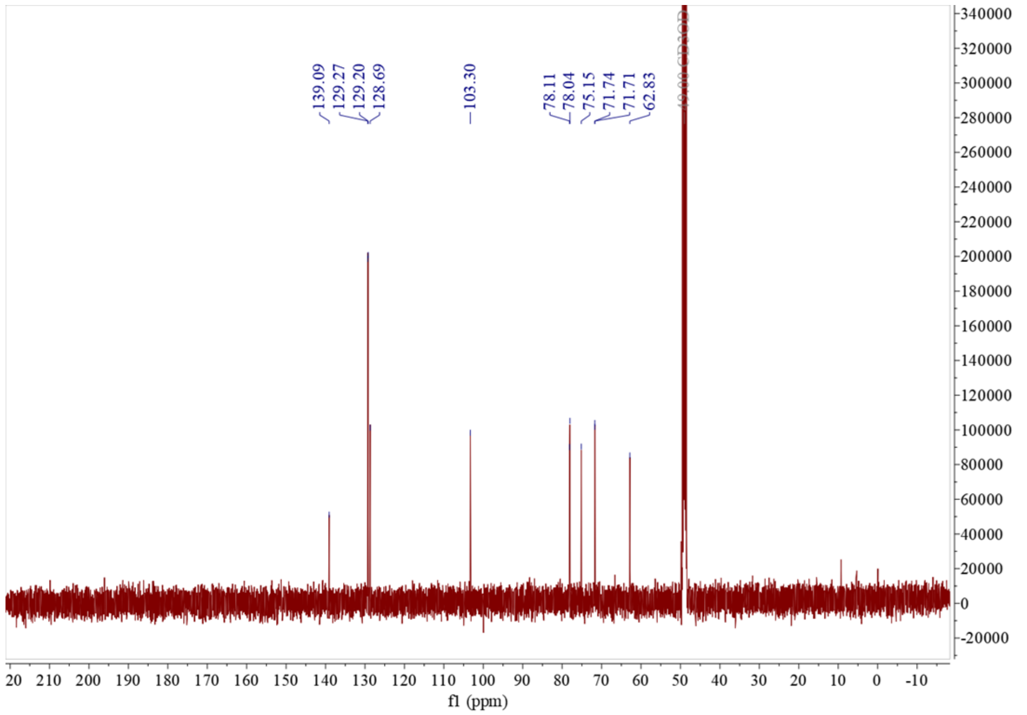

Figure S20 ^{13}C NMR spectrum of **4** in CD_3OD (126 MHz)

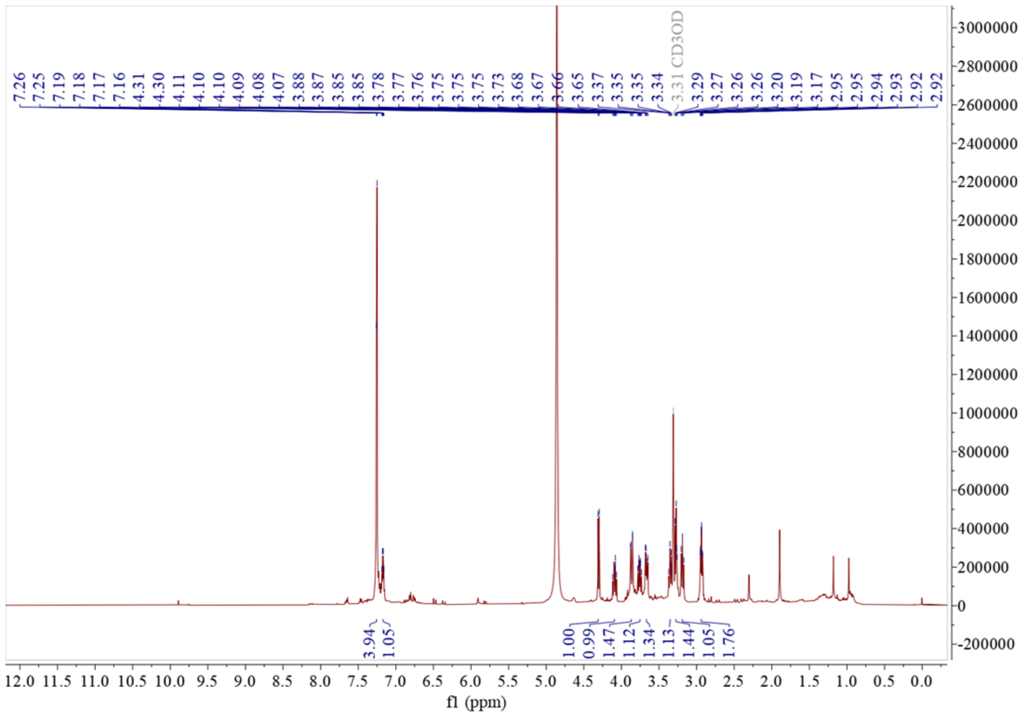

Figure S21 ^{1}H NMR spectrum of **5** in CD_3OD (500 MHz)

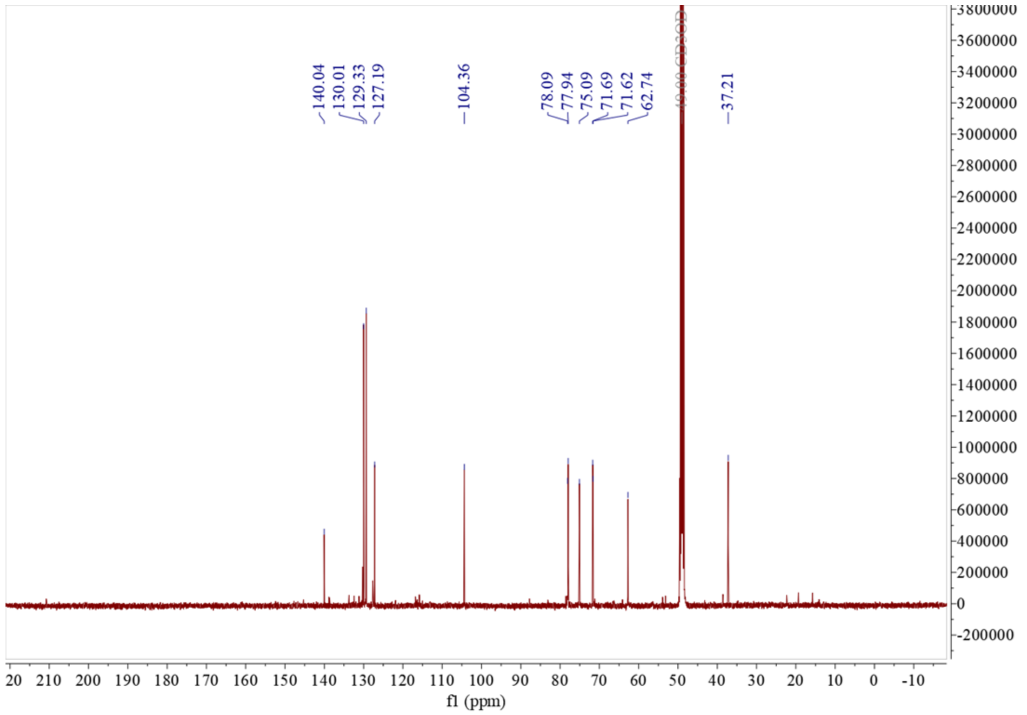

Figure S22 ^{13}C NMR spectrum of **5** in CD_3OD (126 MHz)

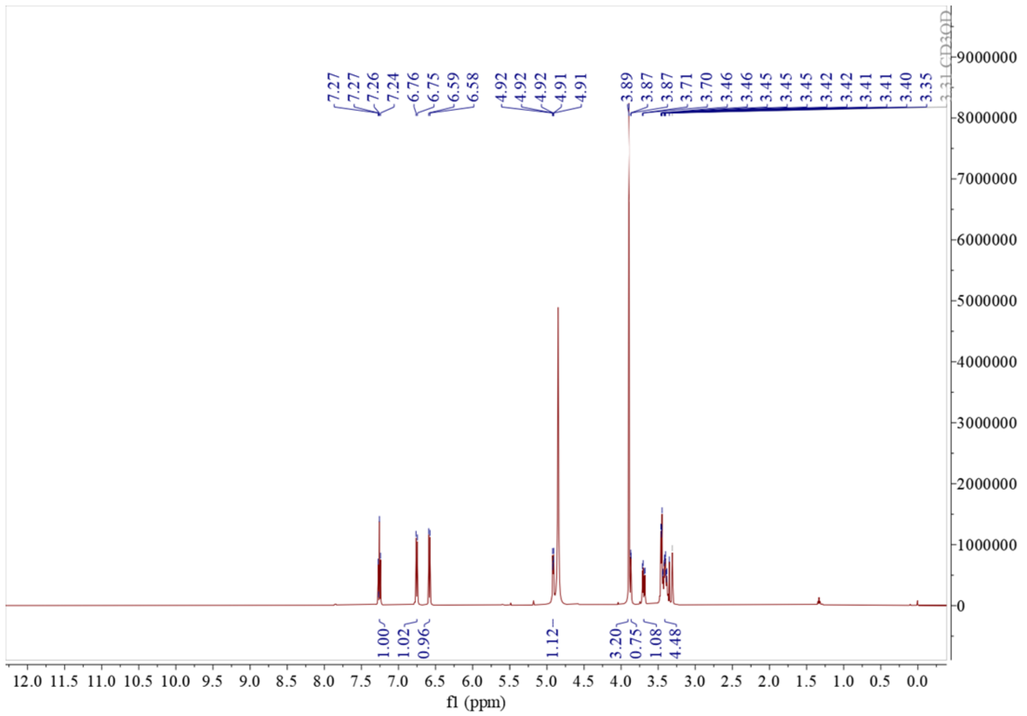

Figure S23 ^{1}H NMR spectrum of **6** in CD_3OD (500 MHz)

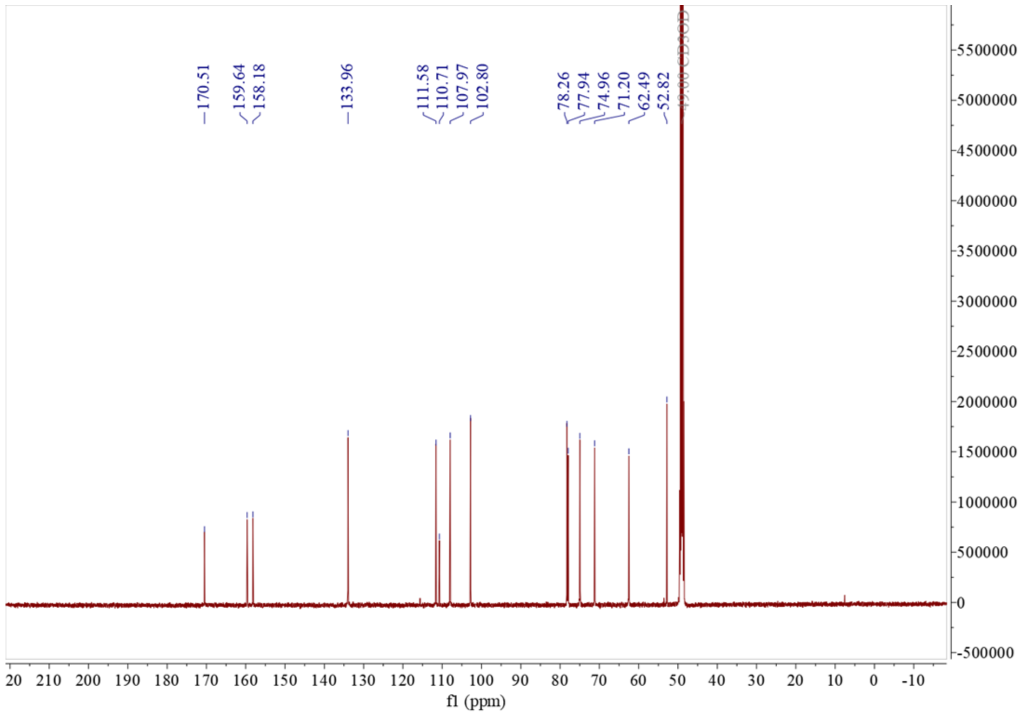

Figure S24 ^{13}C NMR spectrum of **6** in CD_3OD (126 MHz)

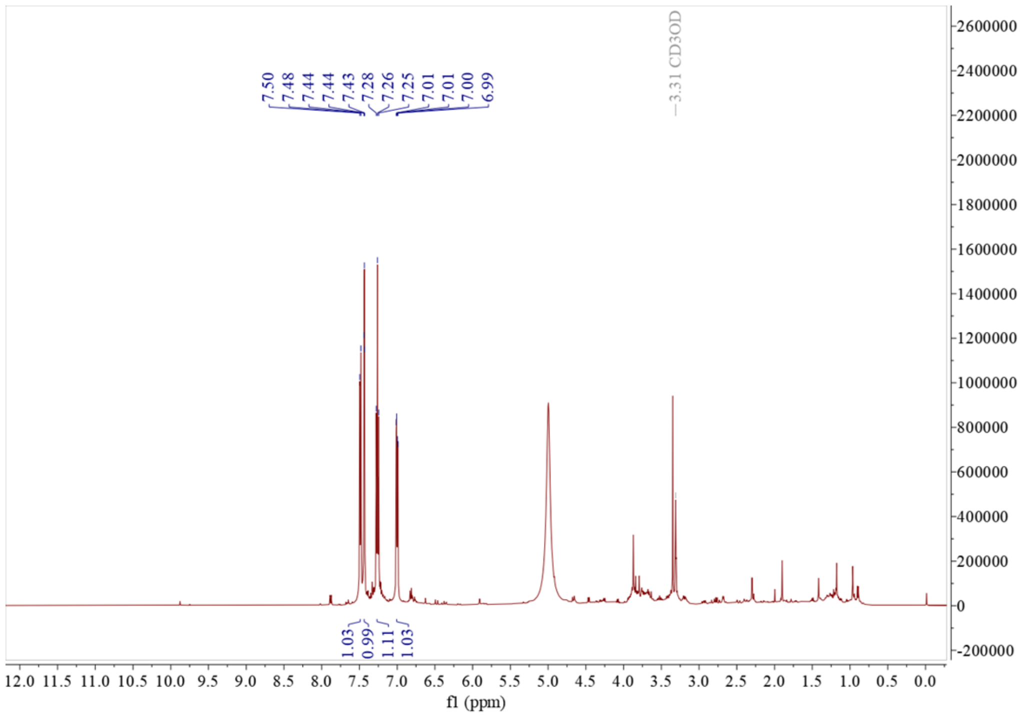

Figure S25 ^{1}H NMR spectrum of **7** in CD_3OD (500 MHz)

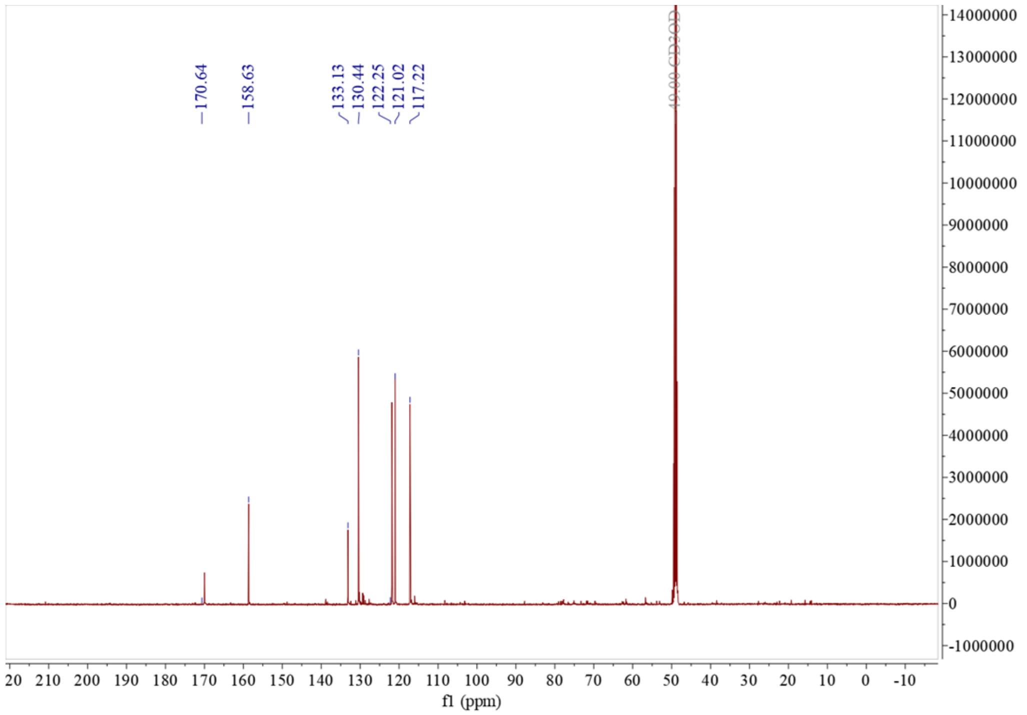

Figure S26 ^{13}C NMR spectrum of **7** in CD_3OD (126 MHz)

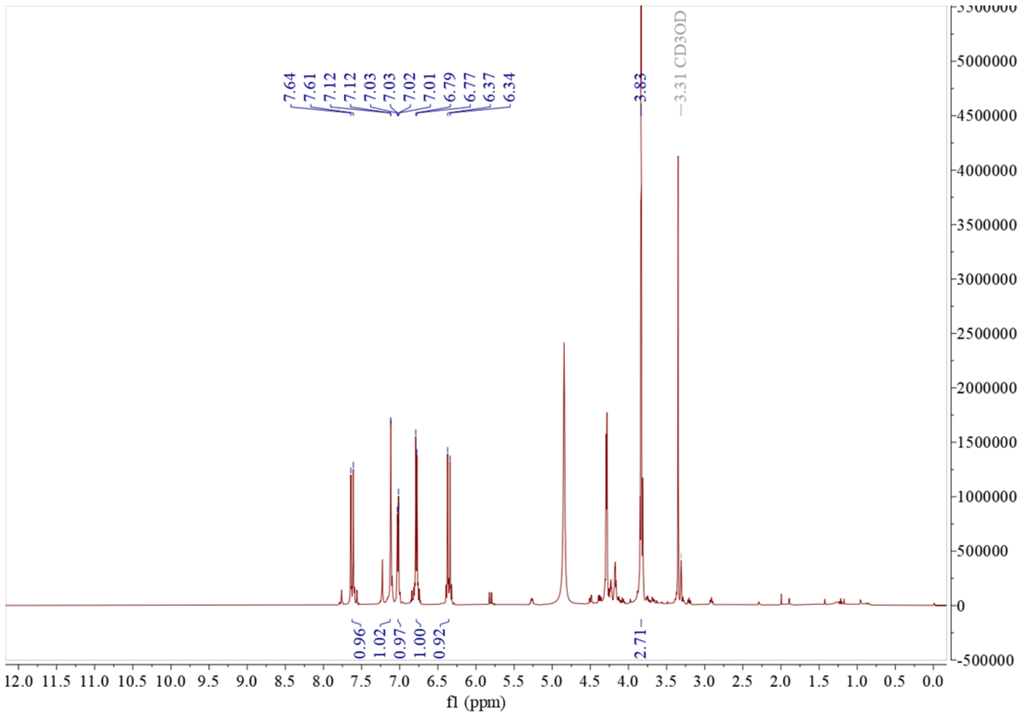

Figure S27 ^{1}H NMR spectrum of **8** in CD_3OD (500 MHz)

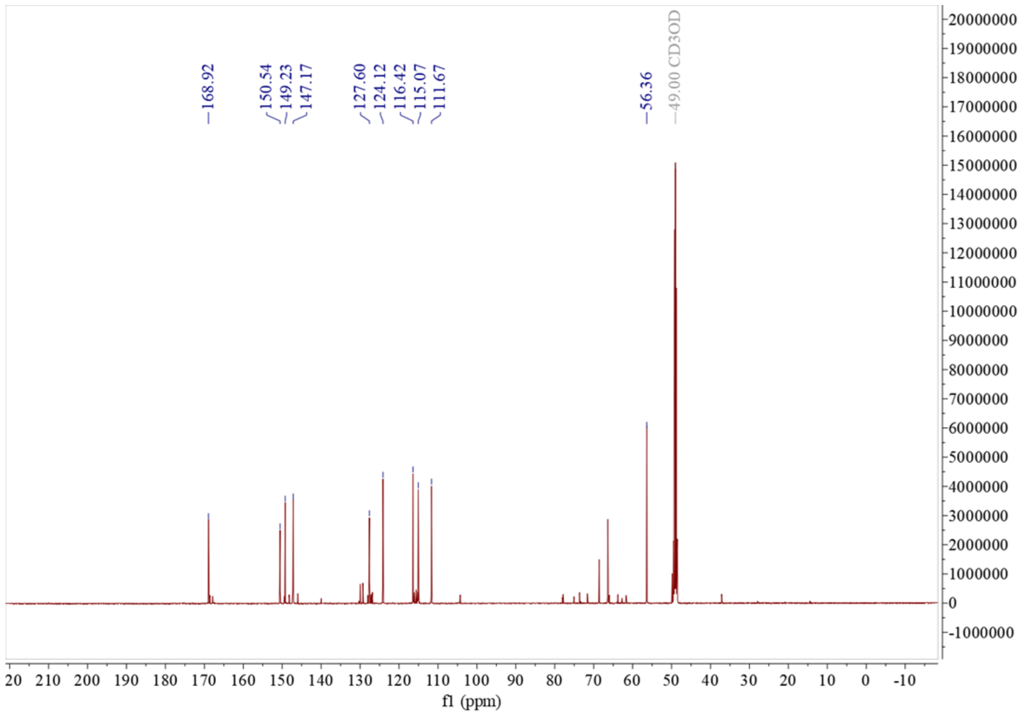

Figure S28 ^{13}C NMR spectrum of **8** in CD_3OD (126 MHz)

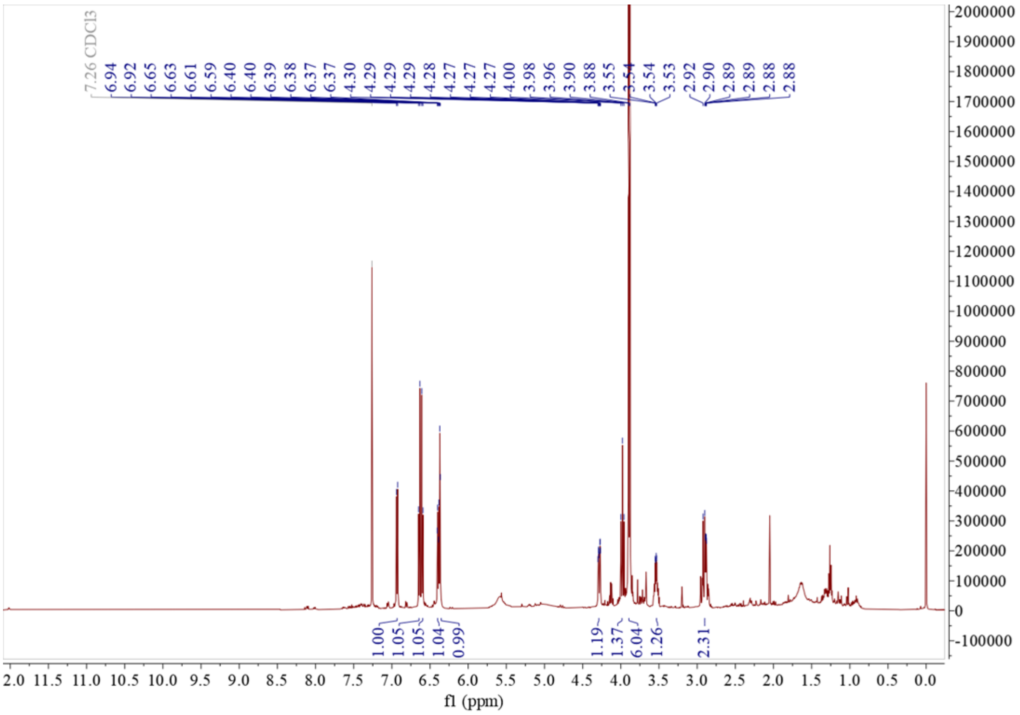

Figure S29 ^{1}H NMR spectrum of **9** in $CDCl_3$ (500 MHz)

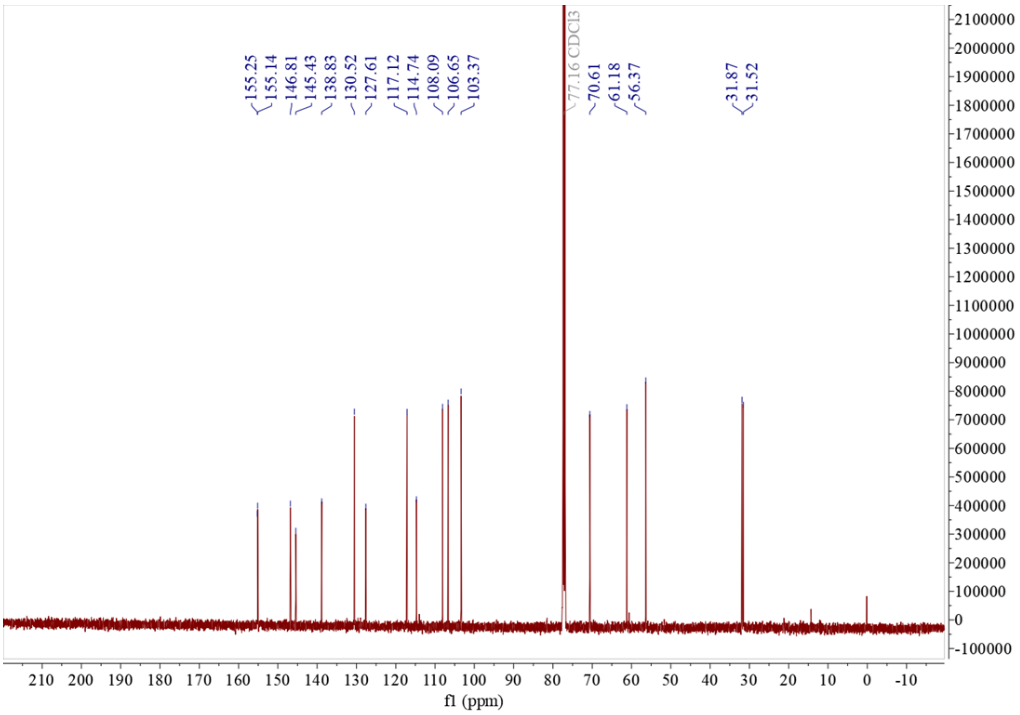

Figure S30 ^{13}C NMR spectrum of **9** in $CDCl_3$ (126 MHz)

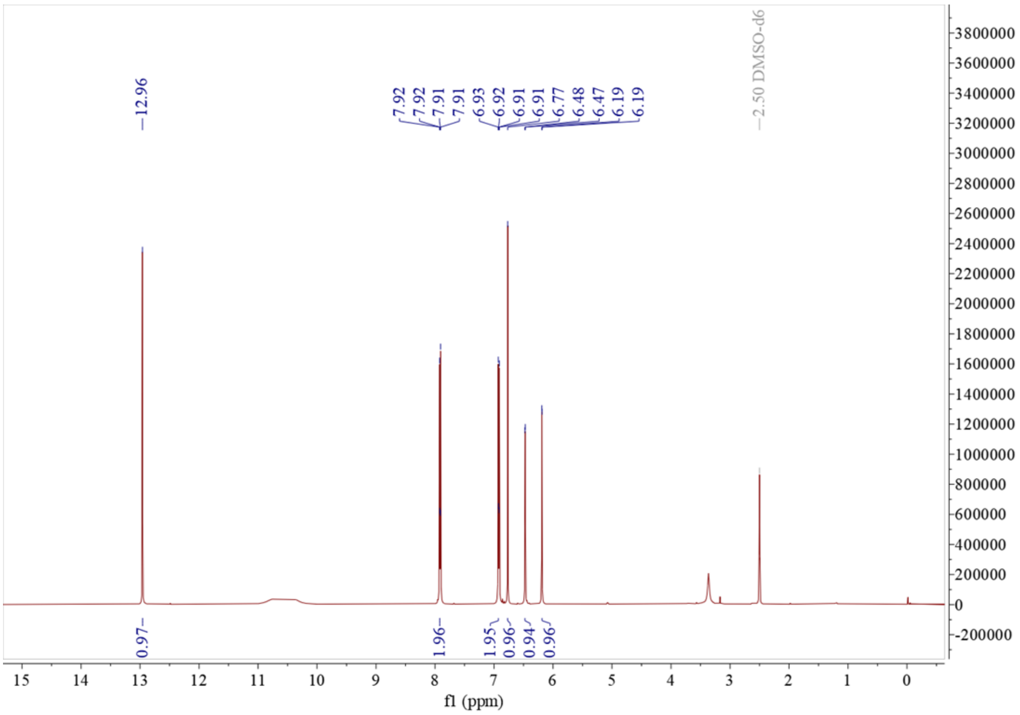

Figure S31 ^{1}H NMR spectrum of **10** in DMSO-*d6* (500 MHz)

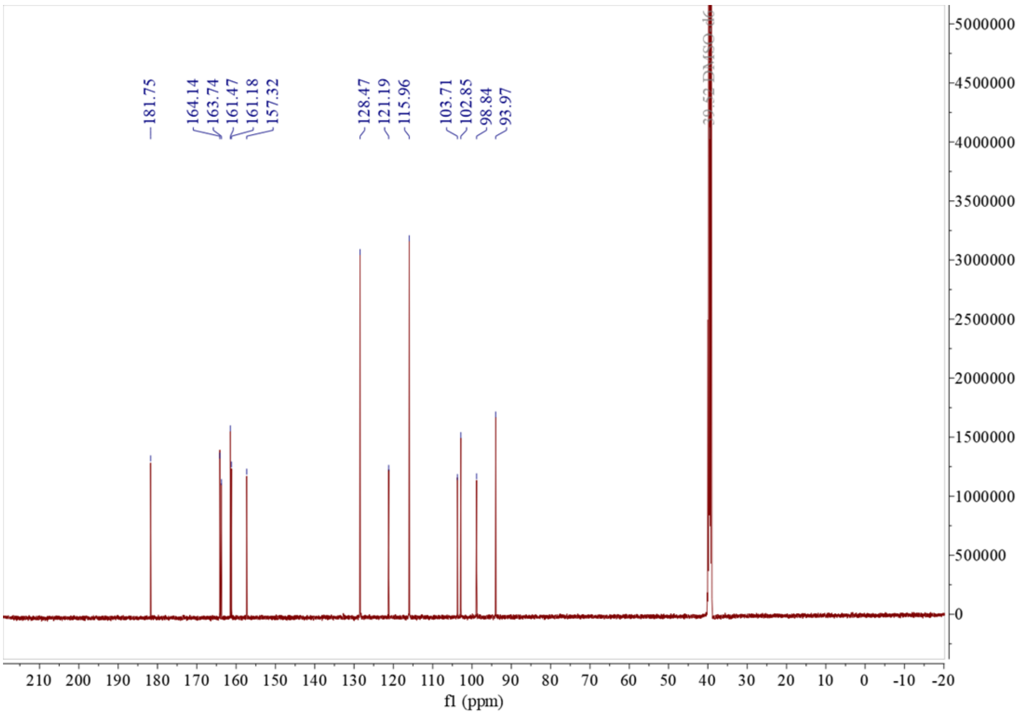

Figure S32 ^{13}C NMR spectrum of **10** in DMSO-*d6* (126 MHz)

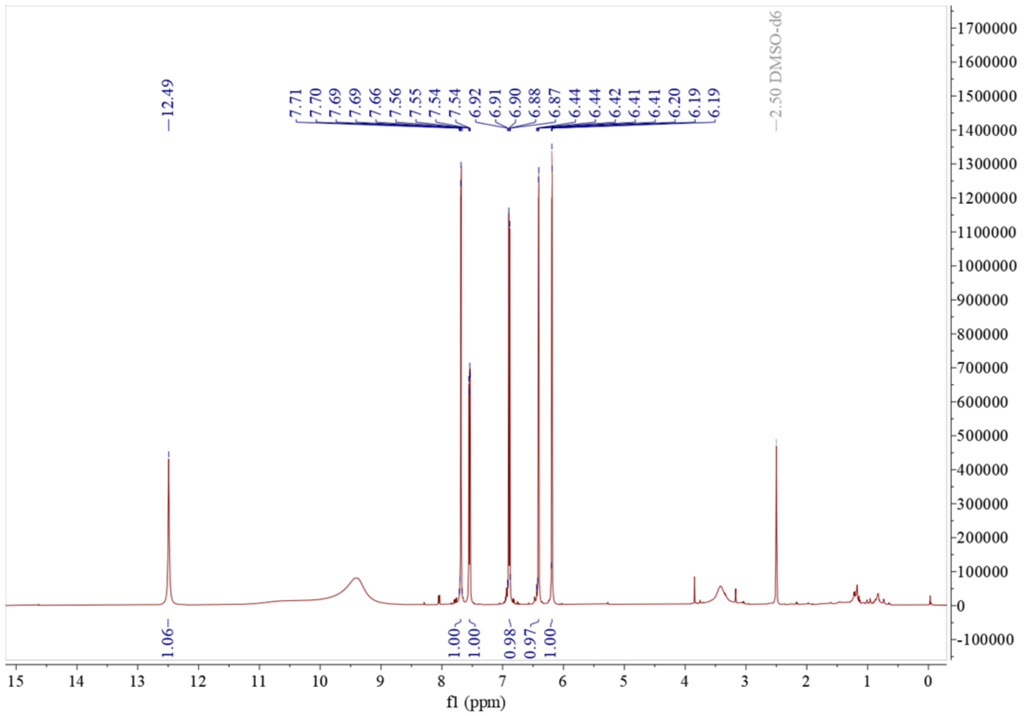

Figure S33 ^{1}H NMR spectrum of **11** in DMSO-*d6* (500 MHz)

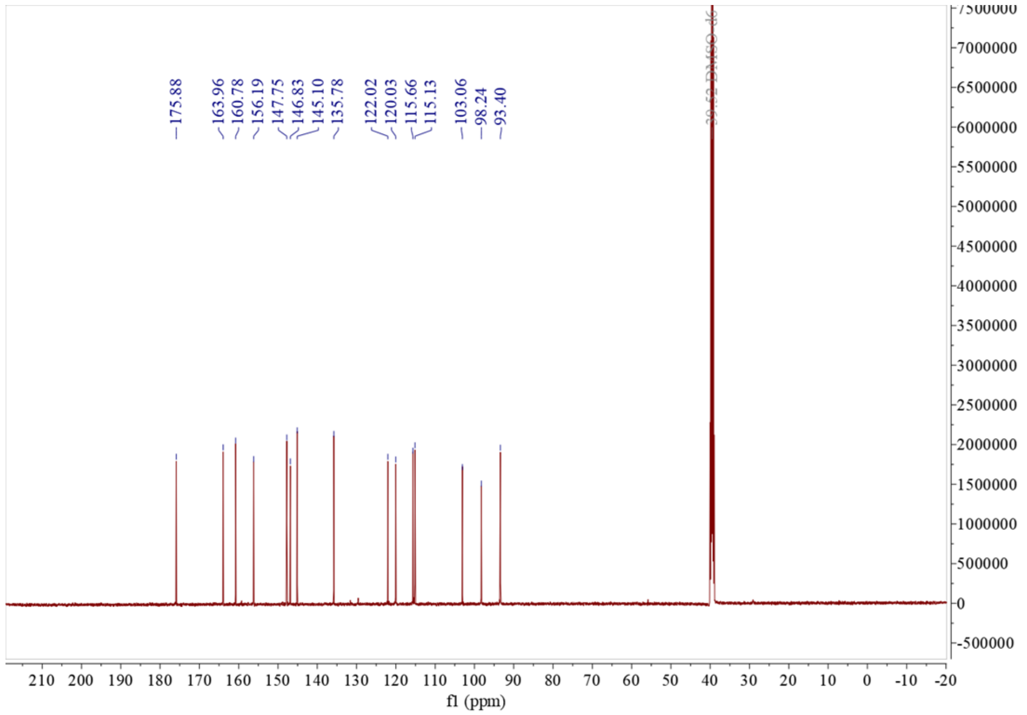

Figure S34 ^{13}C NMR spectrum of **11** in DMSO-*d6* (126 MHz)

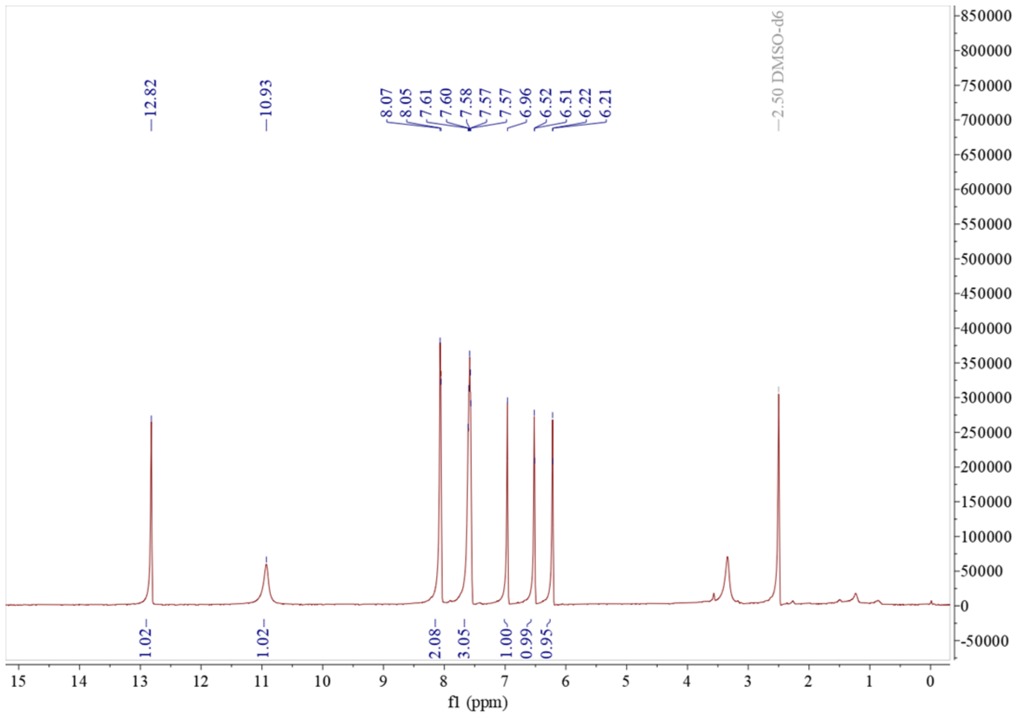

Figure S35 ^{1}H NMR spectrum of **12** in DMSO-*d6* (500 MHz)

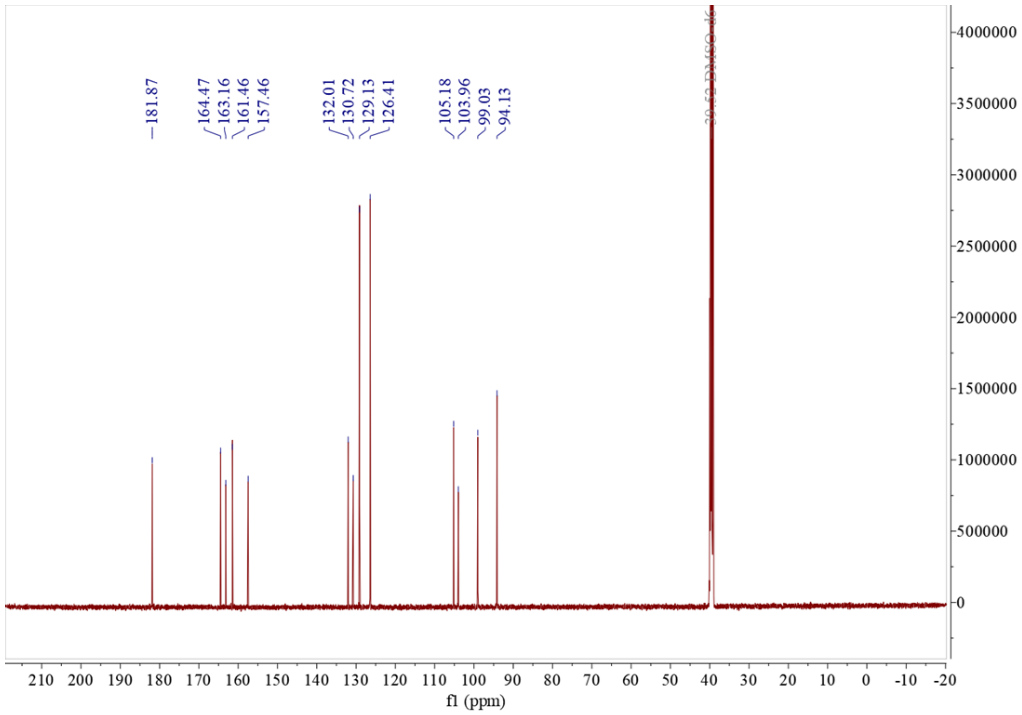

Figure S36 ^{13}C NMR spectrum of **12** in DMSO-*d6* (126 MHz)

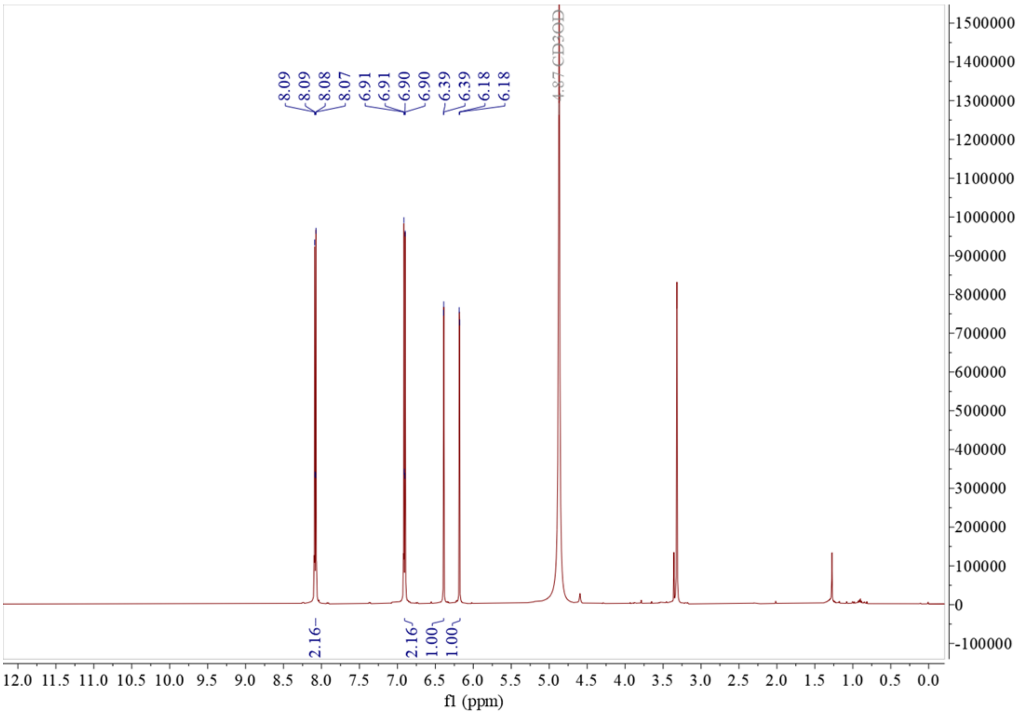

Figure S37 ^{1}H NMR spectrum of **13** in CD_3OD (500 MHz)

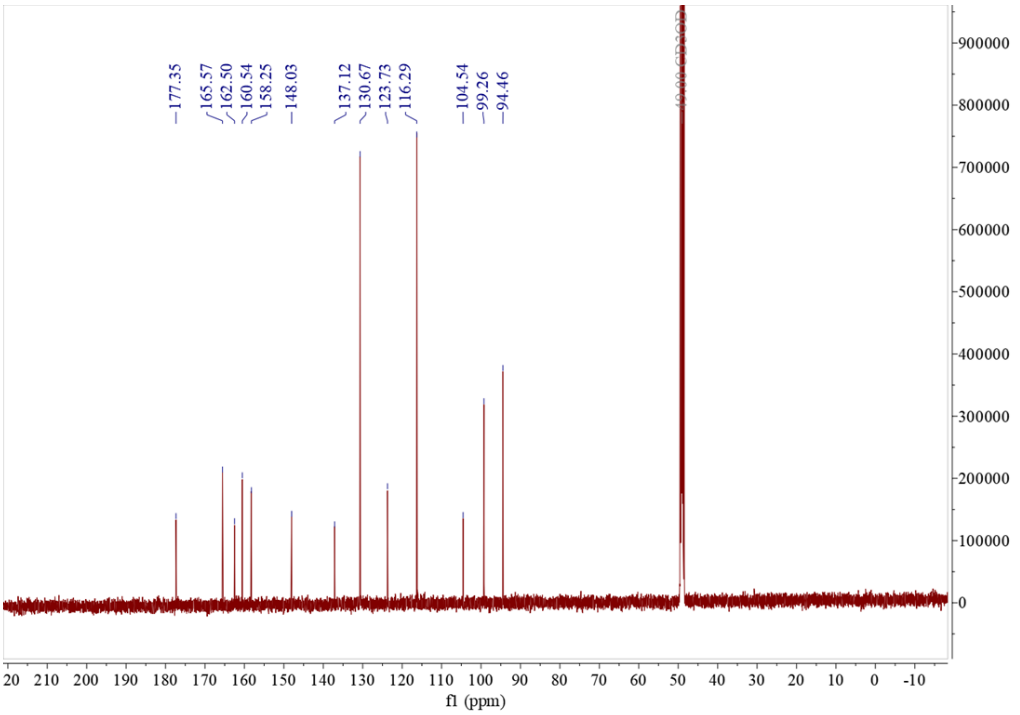

Figure S38 ^{13}C NMR spectrum of **13** in CD_3OD (126 MHz)

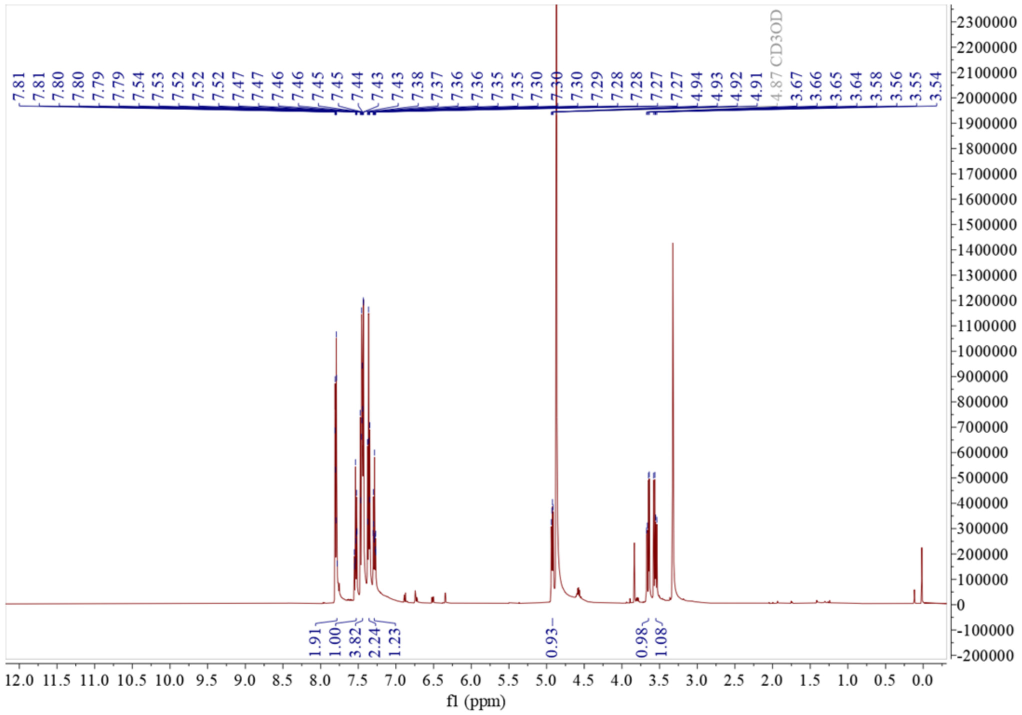

Figure S39 ^{1}H NMR spectrum of **14** in CD_3OD (500 MHz)

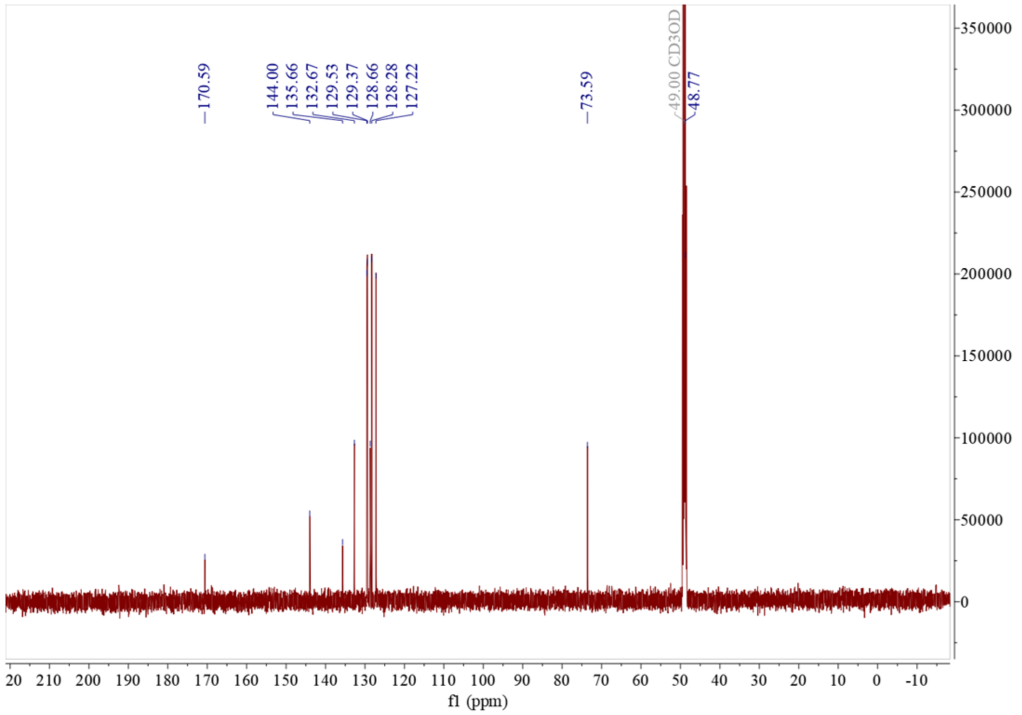

Figure S40 ^{13}C NMR spectrum of **14** in CD_3OD (126 MHz)

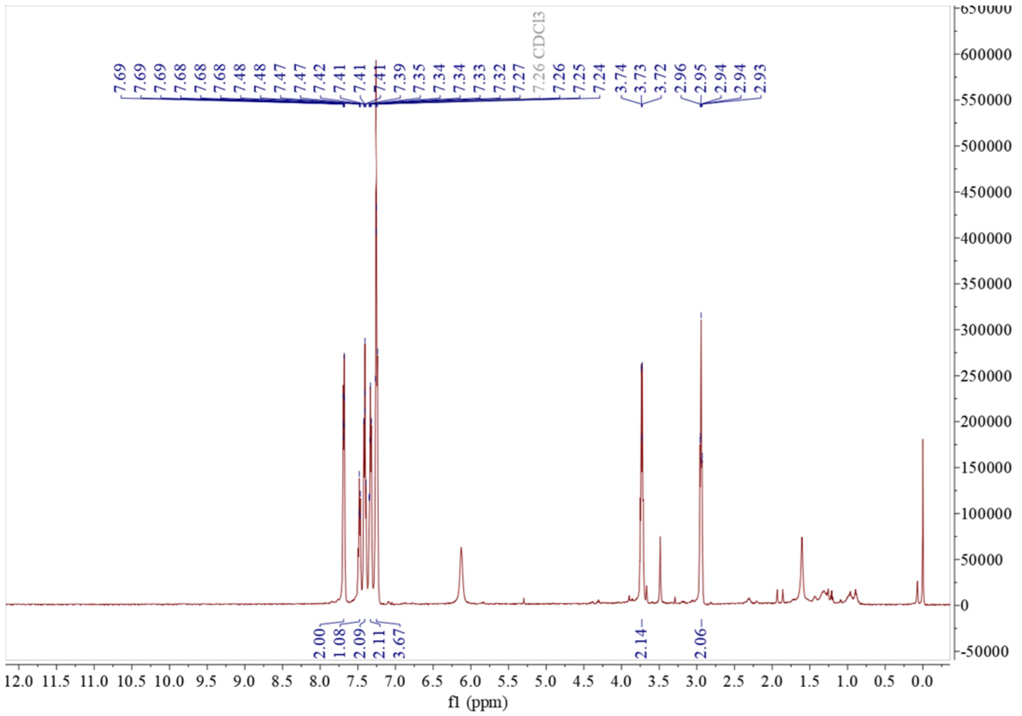

Figure S41 ^{1}H NMR spectrum of **15** in $CDCl_3$ (500 MHz)

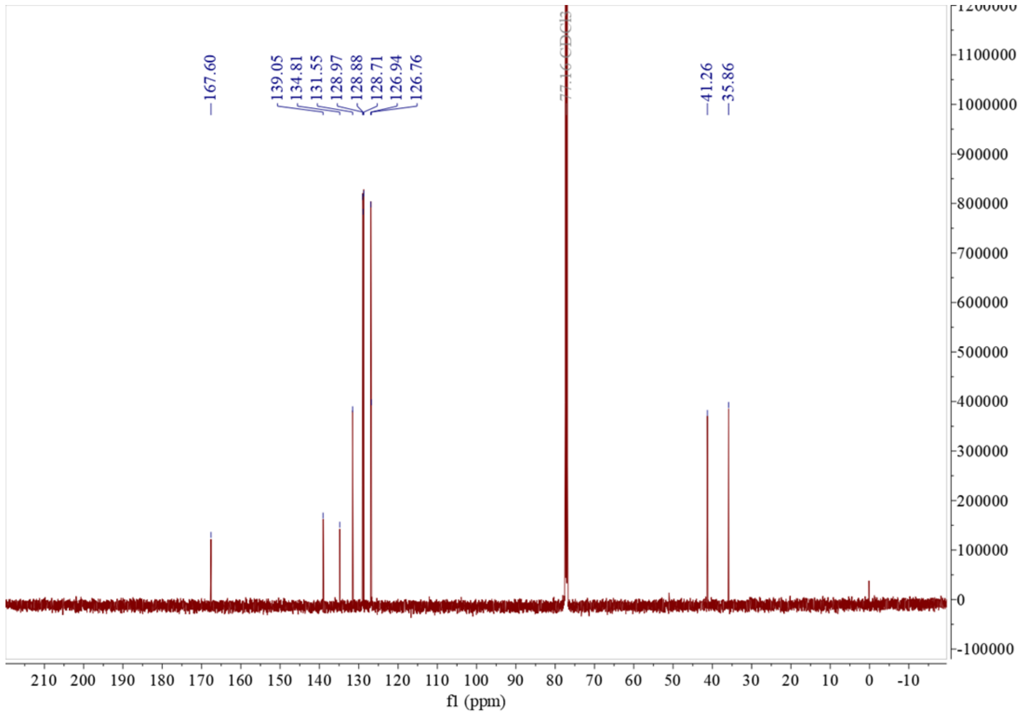

Figure S42 ^{13}C NMR spectrum of **15** in $CDCl_3$ (126 MHz)

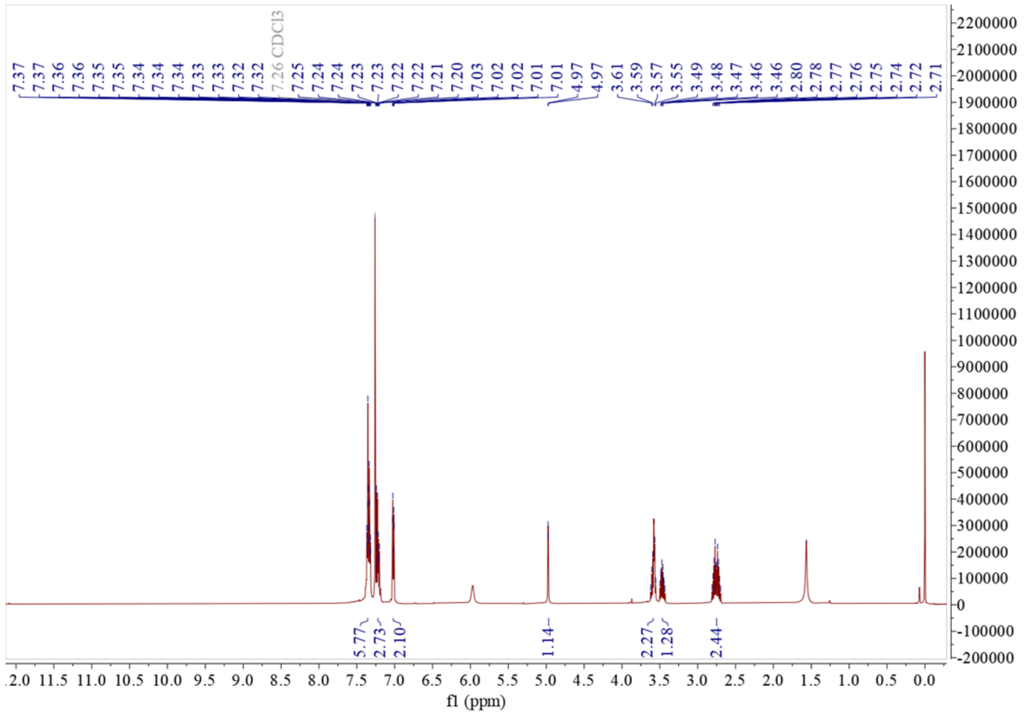

Figure S43 ^{1}H NMR spectrum of **16** in $CDCl_3$ (500 MHz)

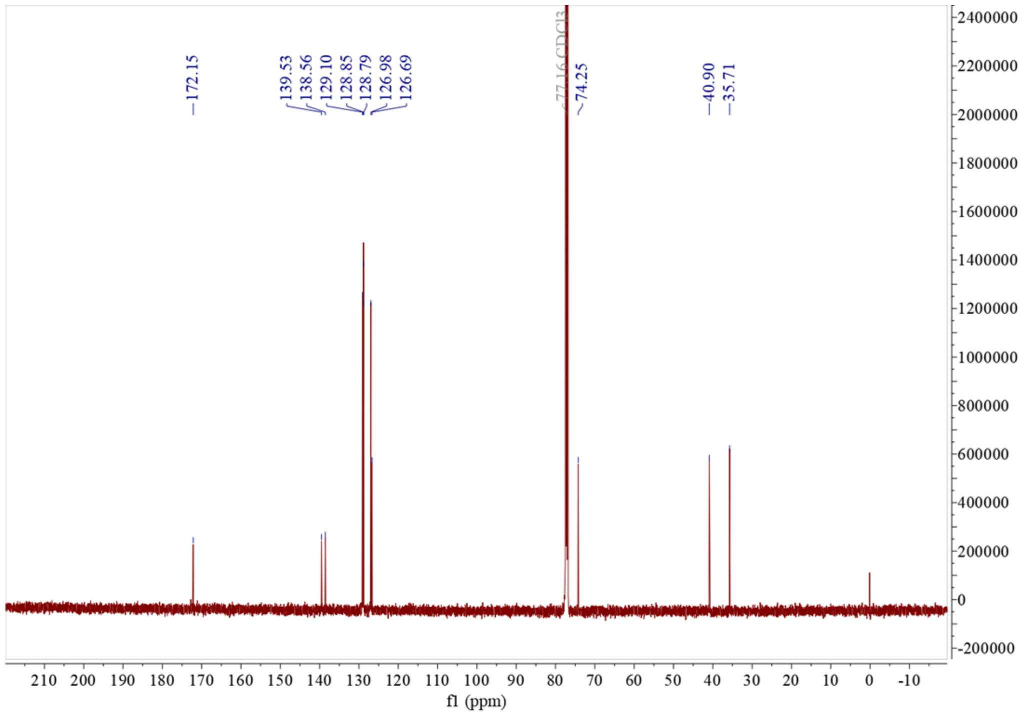

Figure S44 ^{13}C NMR spectrum of **16** in $CDCl_3$ (126 MHz)

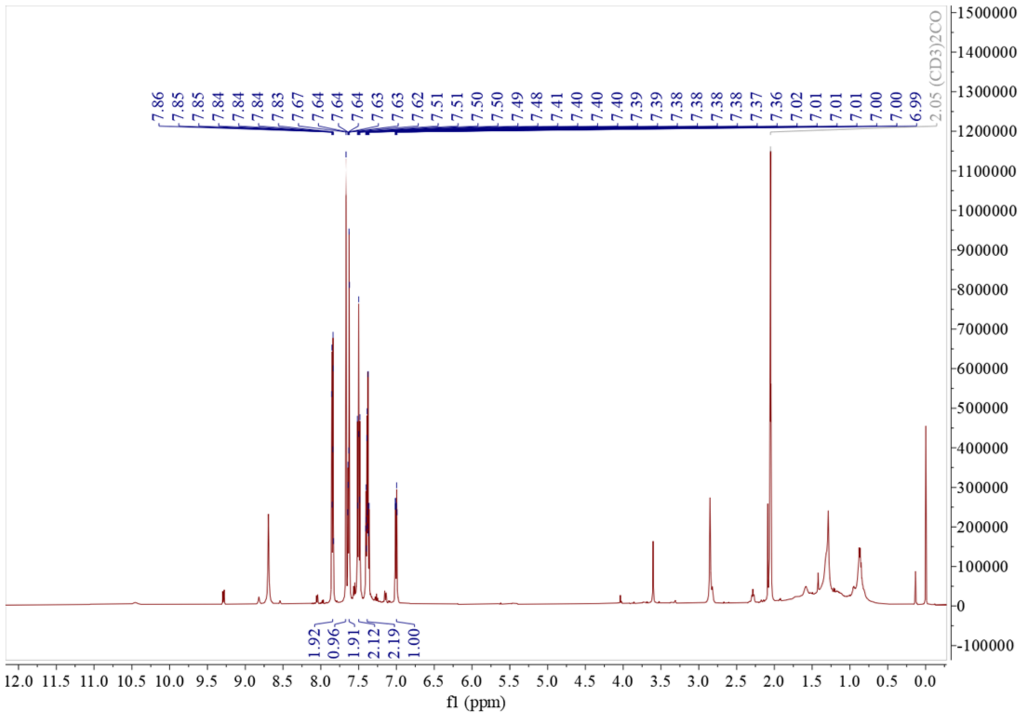

Figure S45 ^{1}H NMR spectrum of **17** in Acetone-*d6* (500 MHz)

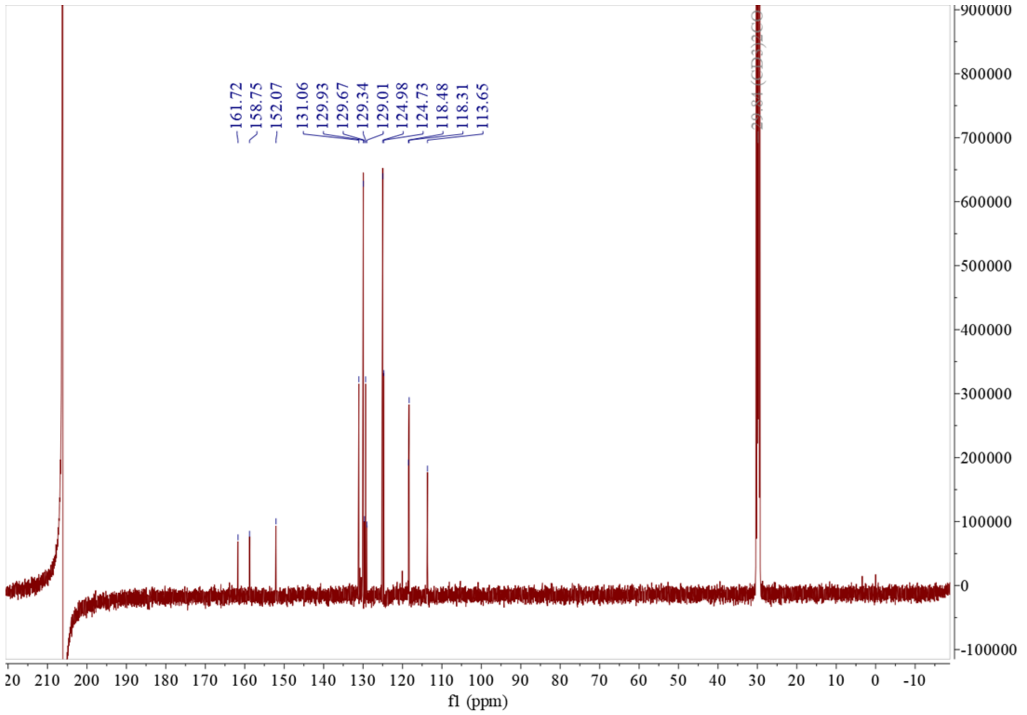

Figure S46 ^{13}C NMR spectrum of **17** in Acetone-*d6* (126 MHz)

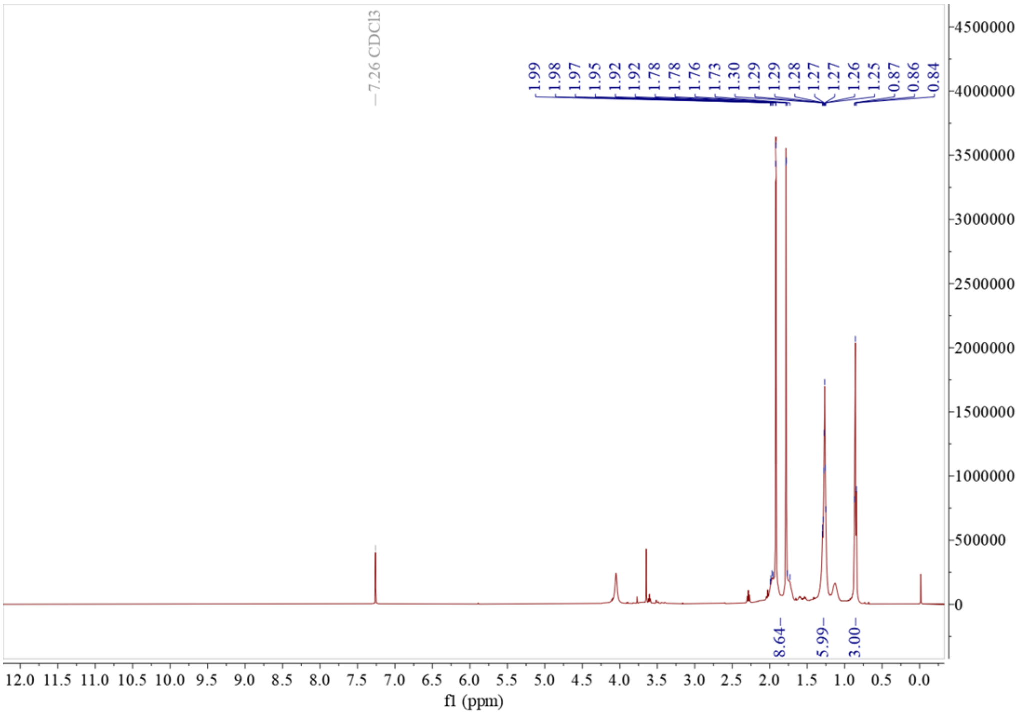

Figure S47 ¹H NMR spectrum of **18** in $CDCl_3$ (500 MHz)

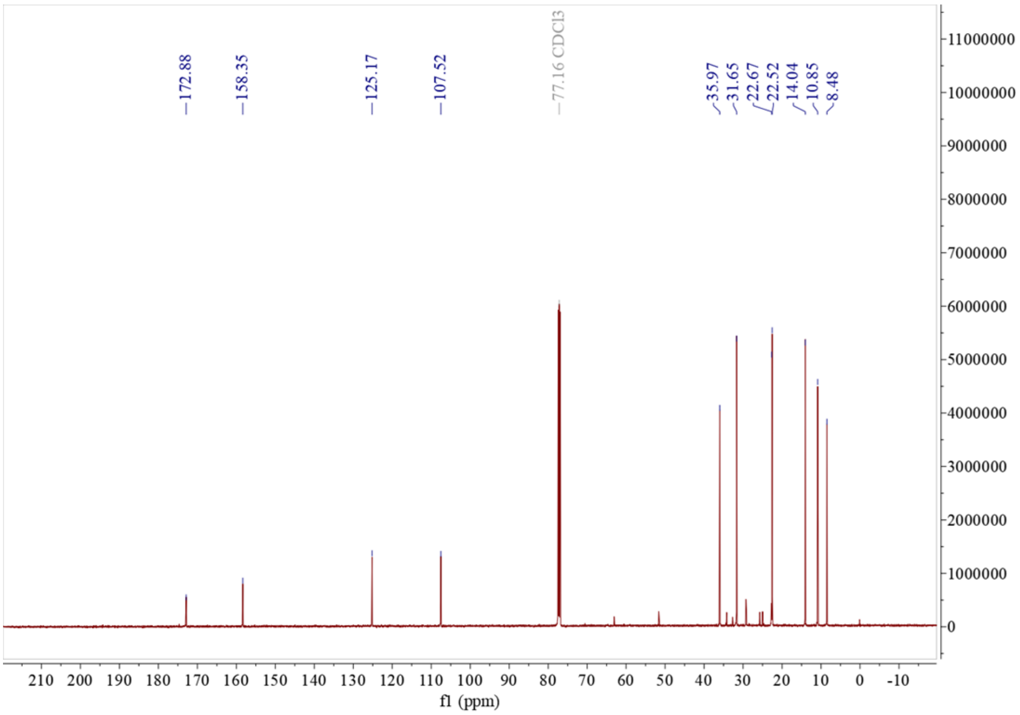

Figure S48 ¹³C NMR spectrum of **18** in $CDCl_3$ (126 MHz)

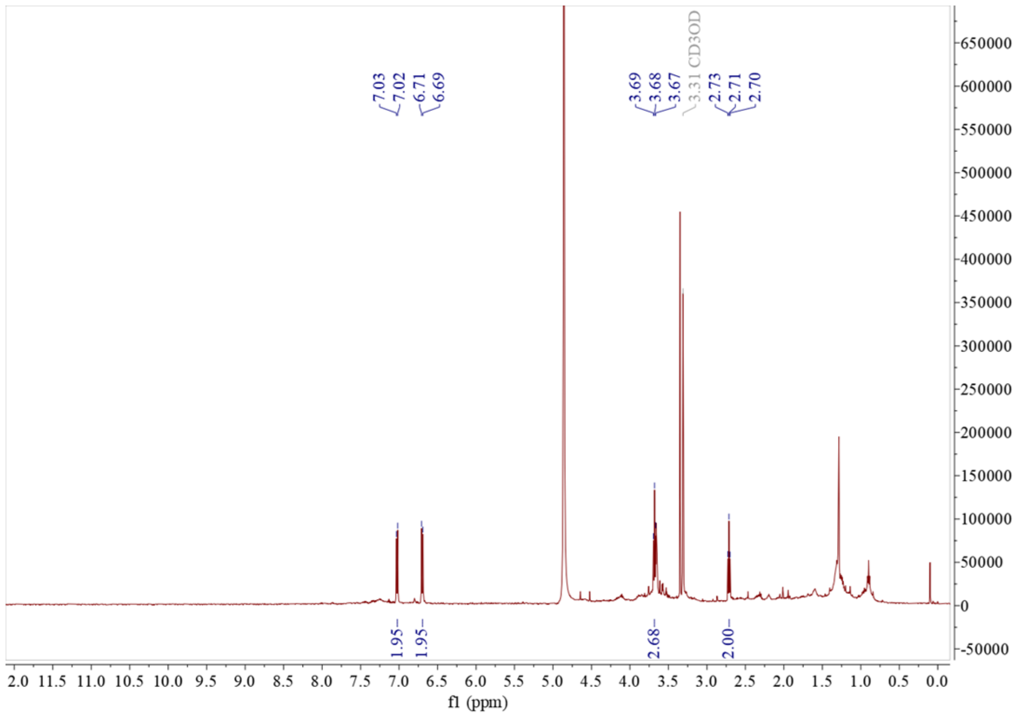

Figure S49 ^{1}H NMR spectrum of **19** in CD_3OD (500 MHz)

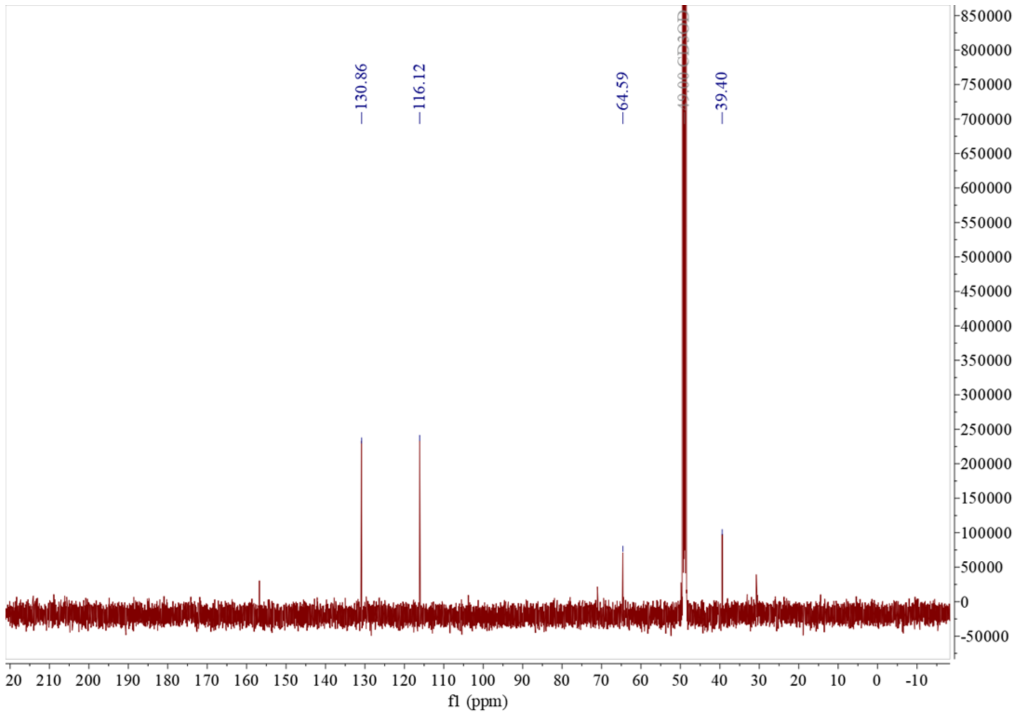

Figure S50 ^{13}C NMR spectrum of **19** in CD_3OD (126 MHz)

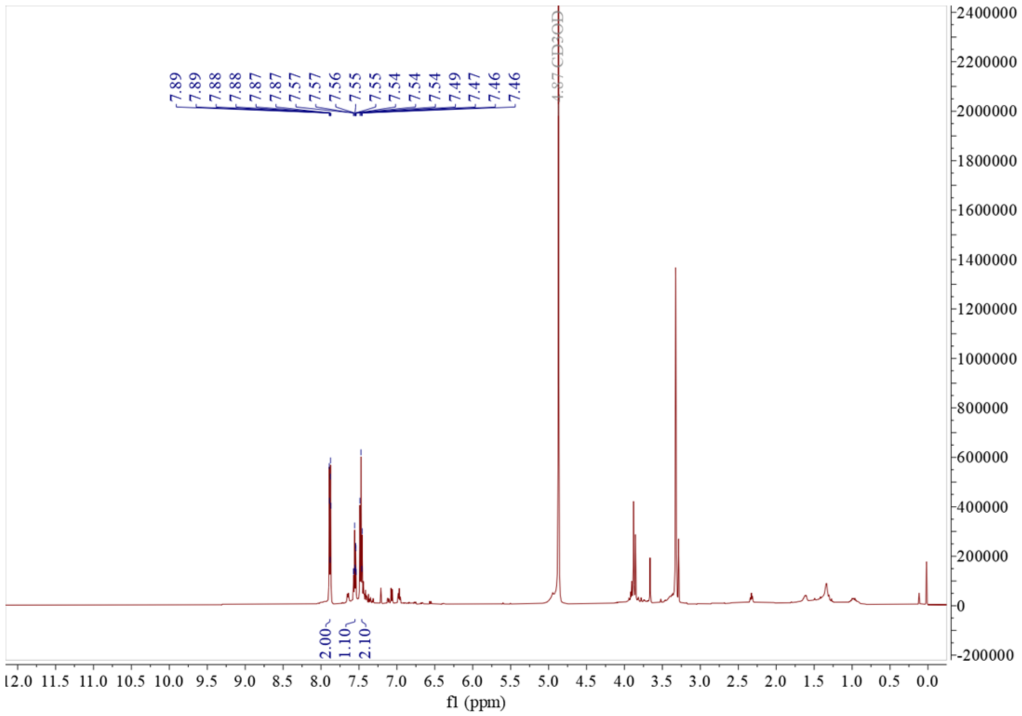

Figure S51 ^{1}H NMR spectrum of **20** in CD_3OD (500 MHz)

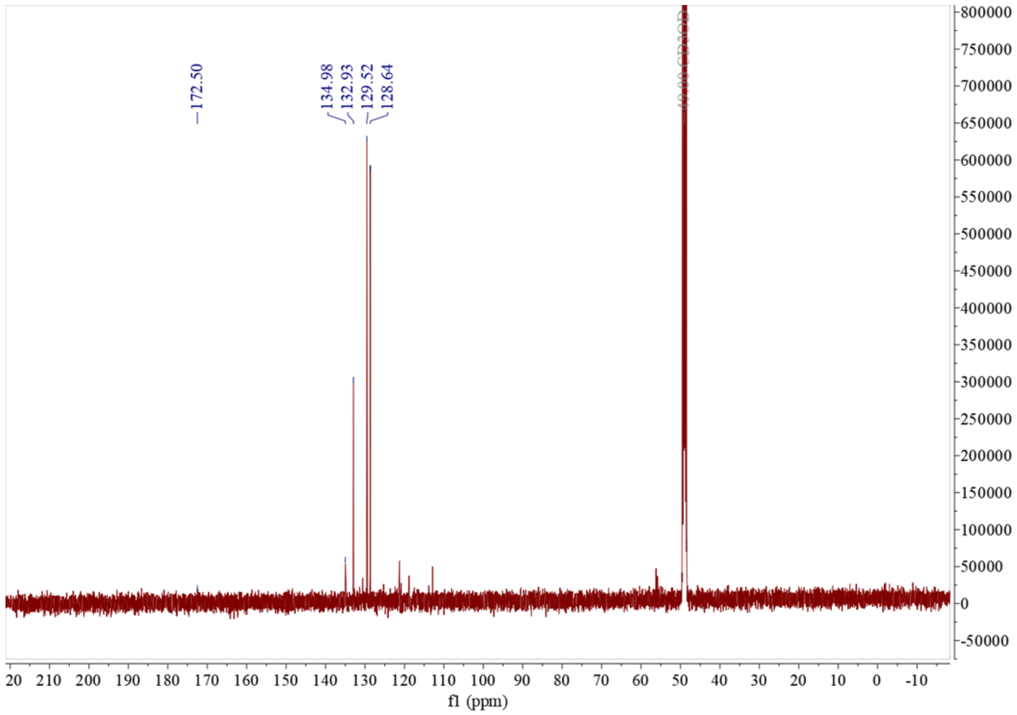

Figure S52^{13}C NMR spectrum of **20** in CD_3OD (126 MHz)

Table S1 ^{1}H NMR (500 MHz) and ^{13}C NMR (126 MHz) data of compounds **2** and **japonicanoside A** (δ in ppm, J in Hz).

	**2**[b]		**japonicanoside A**	
No	δ_H	δ_C	δ_H	δ_C
1		132.8		123.6
2	7.73, t (1.5)	118.4		157.7
3		158.7	7.23 br d(8.4)	117.6
4	7.32, m	122.0	7.43～7.46 overlap	134.6
5	7.43, m	130.8	7.04 br t(7.7)	123.1
6	7.71, dt (1.5, 7.5)	124.3	7.68 dd(7.7，1.6)	131.9
7		167.4		168.0
1'		137.6		137.5
2', 6'	7.48, m	129.2	7.43～7.46 overlap	129.3
3', 5'	7.41, m	129.7	7.35 m	129.5
4'	7.36, m	129.3	7.28 m	129.1
7'	5.36, d (2.5)	67.9	5.30 d(2.8)	67.9
1''	5.10, d, (7.5)	100.2	5.10 d(7.3)	101.2
2''	3.68, m	79.5	3.59～3.64 overlap	80.5
3''	3.61, m	79.1	3.55 m	78.3
4''	3.45, m	71.3	3.33～3.36 overlap	71.2
5''	3.45, m	78.0	3.33～3.36 overlap	77.9
6''	3.72, 3.86, m	62.3	3.59～3.64 overlap，3.79 br d(11.7)	62.5
1'''	5.27, d (1.5)	102.7	5.21 d(1.3)	102.3
2'''	3.95, m	72.2	3.92 m	72.2
3'''	3.60, m	72.2	3.66 m	72.2
4'''	3.40, m	74.0	3.29 m	74.0
5'''	3.97, m	70.0	3.69 m	70.2
6'''	1.28, d (6.5)	18.2	0.95 d(6.2)	17.8

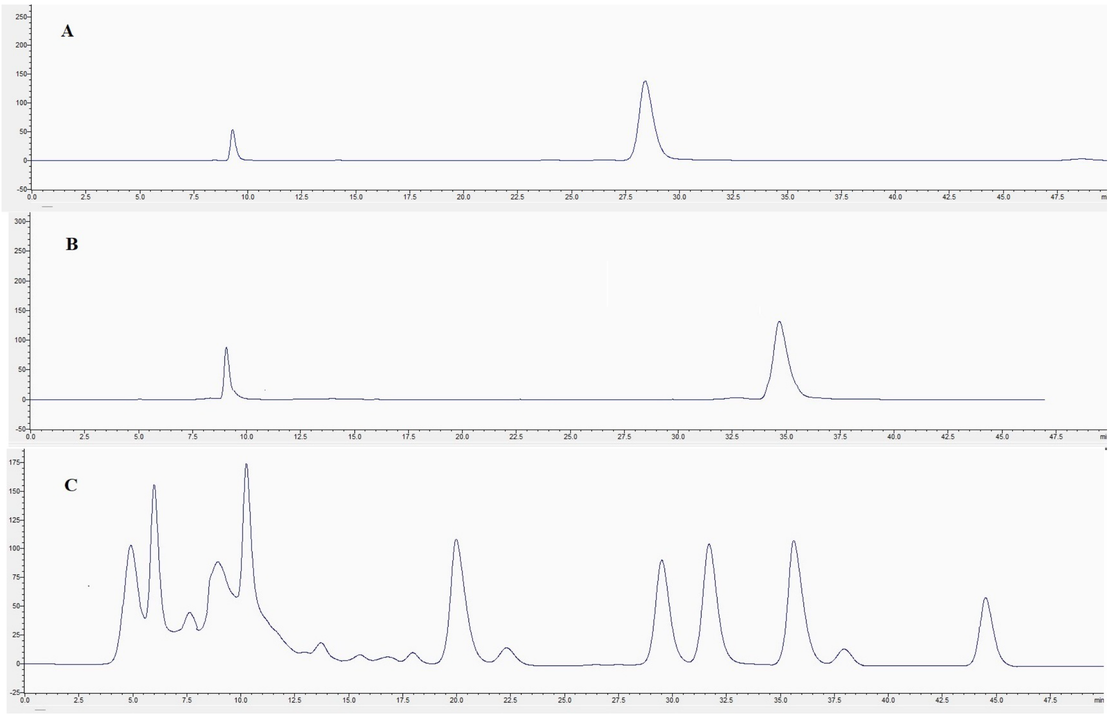

Figure S53 HPLC analyses of the authentic samples (A) D-glucose, (B) L-rhamnose, and (C) the hydrolysates and derivatives of compound **2**

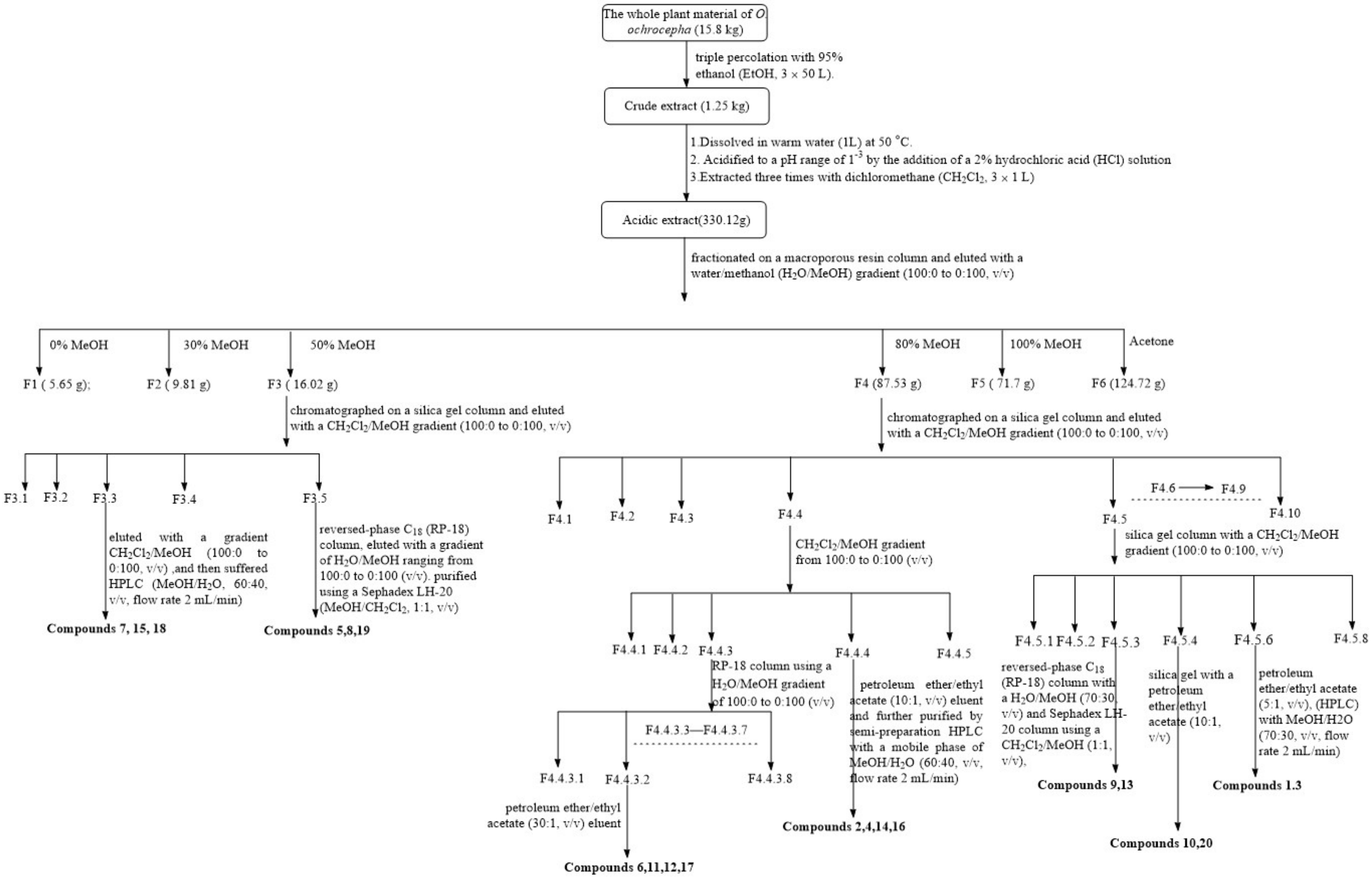

Figure S54 The process of Extraction and Separation
